# Tuning Photoluminescence and Emission Polarization in Metal–Curcumin Complexes via Magnetic Fields

**DOI:** 10.1021/acsomega.6c03000

**Published:** 2026-06-30

**Authors:** Pedro Henrique Dondori Zaramella, Bruno Souza Zanatta, Giovani Bortolini de Oliveira, Fernando Henrique Cristovan, Tatiane Moraes Arantes, Welington de Oliveira Cruz, Raigna Augusta da Silva, Alexandre Marletta, Erick Piovesan

**Affiliations:** 1 Physics Institute, 28119 Federal University of Uberlandia, 38400-902 Uberlandia, Brazil; 2 Institute of Exact Sciences and Technology, 577857 Federal University of Jataí, 75801-615 Jataí, Brazil; 3 Chemistry Institute, 28119 Federal University of Uberlandia, 38400-902 Uberlandia, Brazil

## Abstract

Curcumin complexes with Zn­(II), Pb­(II), and Ca­(II) were synthesized and spectroscopically characterized, with emphasis on magnetic field modulation of photoluminescence and emission polarization. Raman and FTIR analyses confirm coordination through the enolized β-diketone moiety. UV–Vis absorption and photoluminescence spectra exhibit red shifts, 426–432 and 525–534 nm, respectively, indicating metal–ligand interactions and charge-transfer contributions. Vibronic line shape analysis reveals changes in the electronic gap, electron–vibrational mode coupling, and spectral line width, with significant narrowing for the Zn­(II) complex, consistent with increased molecular rigidity. Measurements using the emission ellipsometry technique demonstrate that an external magnetic field modulates both the intensity and polarization of the emitted light of curcumin in a coordination-dependent manner, with the strongest response observed for the Zn­(II) complex. These results show that metal coordination combined with magnetic fields provides an effective strategy to tune photoluminescence and emission polarization in curcumin-based systems, highlighting their potential for photonic and magneto-optical applications.

## Graphical abstract

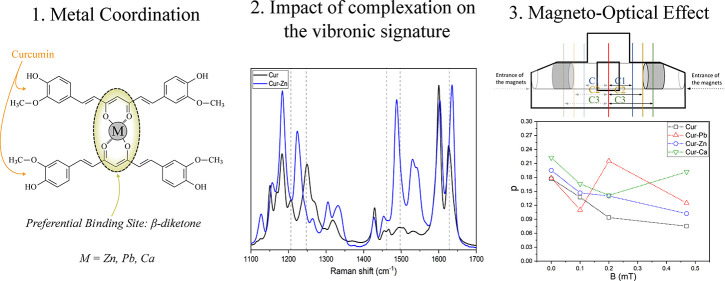

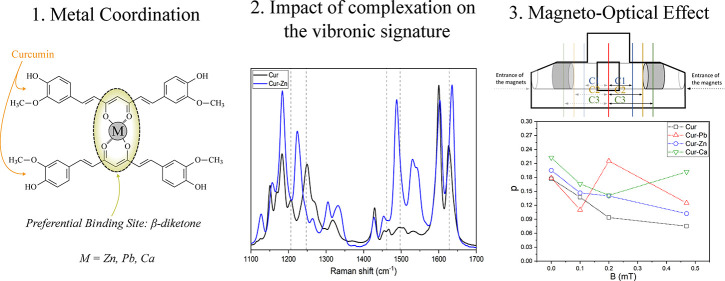

## 1 Introduction

Curcumin is the main bioactive polyphenolic compound extracted from the rhizome of *Curcuma longa*, commonly known as turmeric. The dried and ground rhizome contains approximately 2–8 wt % of curcumin, which has been used for centuries in traditional medicine and food applications.^1–3^ Since its isolation as an active compound, curcumin has attracted considerable scientific interest due to its wide range of pharmacological properties,^4–6^ including antitumor,^8,9^ antimicrobial,^10,11^ anti-inflammatory,^12,13^ antioxidant,^14,15^ hepatoprotective, antiviral, neuroprotective,^16–18^ and antipsoriatic effects.^19–22^ Chemically, curcumin ((1*E*,6*E*)-1,7-bis­(4-hydroxy-3-methoxyphenyl)-1,6-heptadiene-3,5-dione) is characterized by an extended π-conjugated system and the presence of a central β-diketone moiety, which undergoes keto–enol tautomerism (Figure 1).^31^ The molecular structure can be divided into three main units: (A) the β-diketone group, (B) the conjugated heptadienone chain, and (C) the terminal phenolic rings. The conjugation between these structural units is responsible for the intense optical absorption of curcumin in the visible region, as well as for its pronounced sensitivity to the chemical environment.

**Figure 1:**
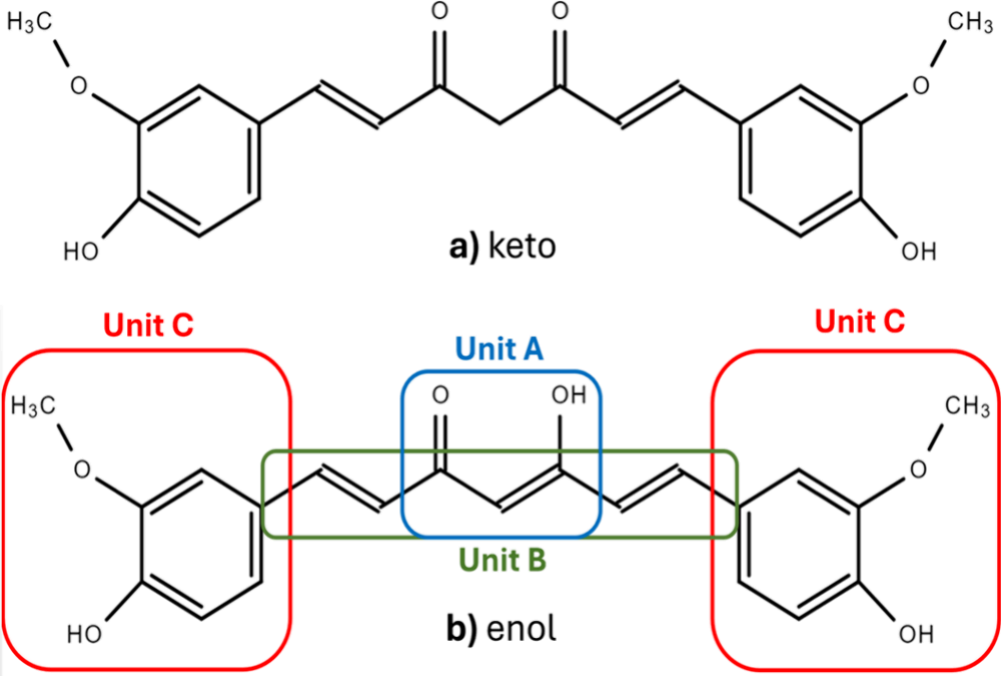
Tautomeric structures of curcumin: (a) keto form and (b) enol form. Units A, B, and C correspond to the β-diketone moiety, the conjugated chain, and the lateral aromatic rings, respectively.

From a coordination chemistry perspective, the β-diketone moiety plays a central role. In its enolized form, this group provides an efficient chelation site for metal ions through oxygen donors, enabling the formation of stable metal–curcumin complexes.^2,3,21,22^ Several studies have demonstrated that coordination with transition or alkaline-earth metals can significantly modify the physicochemical properties of curcumin, including its solubility, stability, electronic structure, and biological activity.^3,7,21,23^ Recent studies have further refined the understanding of curcumin–metal coordination, particularly through combined vibrational and structural analyses, confirming the sensitivity of the β-diketone moiety to metal binding and its impact on molecular stability and electronic distribution.^24,25^ These modifications arise primarily from changes in the conjugation length, charge distribution, and conformational flexibility induced by metal binding. Beyond its biological relevance, curcumin has also emerged as a versatile functional material for optical and sensing applications. The role of metal coordination in tuning charge-transfer interactions and excited-state dynamics in curcumin-based systems leads to controlled modulation of optical absorption and emission properties.^26^ Its strong absorption, efficient photoluminescence, and sensitivity to molecular interactions have enabled its use in chemical sensors, metal-ion detection, and optoelectronic systems.^9,10,27–29^ In this context, spectroscopic techniques such as UV–vis absorption, Raman, Fourier transform infrared (FTIR), and photoluminescence spectroscopy provide powerful tools for probing the effects of metal coordination on the electronic and vibrational structure of curcumin. Complementary spectroscopic approaches have also been employed in recent studies to correlate structural modifications with optical responses in metal–organic systems, reinforcing the importance of multitechnique analysis in these materials.^30^

In this work, we report the synthesis and detailed spectroscopic characterization of curcumin complexes with Zn­(II), Pb­(II), and Ca­(II). Raman and FTIR spectroscopies are used to elucidate the coordination mechanism, while UV–vis absorption and photoluminescence measurements probe metal-induced modifications in the electronic structure. Vibronic photoluminescence line shape simulations based on Fermi’s Golden Rule and Franck–Condon analysis are used to quantify changes in electronic gap, electron–vibrational modes coupling, and spectral broadening. Furthermore, magnetic field effects on polarization-dependent emission are investigated using an emission ellipsometry-based technique.^55^ Finally, the novelty of the present study is the use of emission ellipsometry under an external magnetic field to probe these effects, providing a comprehensive picture of how metal coordination modulates the optical and magneto-optical properties of curcumin, enabling the development of magneto-optical probes.

## 2 Materials and Methods

Curcumin (no. C1386-10G) was purchased from Sigma-Aldrich, while calcium, lead, and zinc acetates were obtained from Êxodo Científica. All reagents were used as received, without further purification. Since curcumin exhibits poor solubility in aqueous media,^32^ ethanol was employed as the solvent. A solution of curcumin (239 mg in 15 mL of ethanol, 0.65 mmol) was prepared at 60 °C under continuous stirring for 3 h, following a previously reported procedure.^33^ The resulting solution was stored under refrigeration and protected from light to prevent photodegradation.^32^ For the synthesis of the metal complexes, the curcumin solution and the corresponding metal acetate solution (0.65 mmol in 1 mL of ethanol) were preheated separately to 60 °C, under stirring. The curcumin solution was then added dropwise to the metal acetate solution over a period of at least 15 min, after which the mixture was refluxed for 3 h at 60 °C under constant stirring. The reaction mixture was subsequently filtered, and the resulting precipitate was washed three times with cold ethanol (4 °C) to ensure the complete removal of the supernatant. The solid product was dried in an oven at 60 °C to remove residual solvent and then stored in a desiccator to prevent moisture uptake. The other complexes were synthesized using similar procedures.

Aliquots of the powdered samples were dissolved in dimethyl sulfoxide (DMSO) for further analysis. Dissolution was performed at 60 °C under continuous stirring for 1.5 h. In total, four samples were obtained: curcumin (Cur), curcumin–calcium complex (Cur–Ca), curcumin–lead complex (Cur–Pb), and curcumin–zinc complex (Cur–Zn). These samples were analyzed both in the solid state and in solution for direct comparison. A photograph of the powdered samples is presented in Figure SI0 of the Supporting Information.

Optical absorption spectra were recorded over the 300–1100 nm range with 2 nm steps using a FEMTO 800 XI spectrophotometer. Photoluminescence (PL) and ellipsometry measurements were performed using an iZi diode laser (405 nm) in combination with an Ocean Optics USB4000 spectrophotometer. For the ellipsometry setup, a custom-built sample holder was fabricated using 3D printing technology, specifically designed to accommodate magnets for magnetic field–dependent measurements. The experimental setup is shown in Figure SI1 (Supporting Information), and Figure SI2 illustrates the 3D-printed sample holder used for magnet-based experiments. The magnetic fields were measured using a Hall probe positioned at the center of the sample holder, corresponding to the position of the cuvette. Table 1 summarizes the distances between the magnets and the samples, as well as the corresponding magnetic field strengths measured experimentally.

**Table 1:** Distances and Magnetic Field Values Correspond to Each Magnet Position in the Sample Holder

label	distance (cm)	magnetic field (mT)
C1	0.5	0.47
C2	1.0	0.20
C3	1.5	0.10

Raman spectra were acquired using a LabRAM HR Evolution spectrometer (Horiba) equipped with a 785 nm excitation laser line. Fourier transform infrared (FTIR) spectra were recorded with a Cary 630 FTIR spectrometer (Agilent), in transmission mode using KBr pellets, with 16 accumulations and a spectral resolution of 4 cm^–1^.

The PL spectrum simulation was performed using the *Line Shape Luminescence Spectrum* program, which is based on the electronic–vibrational transition rate, Fermi’s Golden Rule, using the adiabatic and Condon approximations. Considering the electronic transition from the excited-state *b* to the ground state *a* (*b* → *a*), the transition probability can be expressed as follows:^34,35^

$$I\left(\omega \right)=\frac{2a_{m}\pi \omega }{3c\hbar }{\left|{\mathrm{\mu ⃗}}_{ab}\right|}^{2}\int_{-\infty }^{+\infty }dt\;e^{\left(it\left(\omega_{ab}-\omega \right)-d^{2}t^{2}/2\right)}\underset{j}{\prod }G_{j}^{*}\left(t\right)$$ 1

where μ⃗_ab_ is the electronic transition moment, *a*_m_ accounts for medium effects, *c* is the speed of light, *ℏ*ω_*ab*_ = *E*_*b*_ – *E*_*a*_ is the energy difference between the lowest unoccupied molecular orbital (LUMO) and highest occupied molecular orbital (HOMO) electronic states, and *d* represents the inhomogeneous spectral broadening, described by a Gaussian distribution. The function *G*_j_^*^(*t*) corresponds to the Franck–Condon factor in the harmonic approximation, which in the case of pure displacement can be expressed as^34,35^

$$\underset{j}{\prod }G_{j}^{*}\left(t\right)=e^{-\sum_{j=1}^{N}S_{j}\left[\left({ñ}_{j}+1\right)e^{it\omega_{j}}+{ñ}_{j}e^{-it\omega_{j}}-\left(2{ñ}_{j}+1\right)\right]}$$ 2

where *S*_*j*_ is the Huang–Rhys factor, ω_*j*_ is the *j*th phonon energy, ${ñ}_{j}=\frac{1}{\left(e^{\hbar \omega_{j}/k_{B}T}-1\right)}$ is the vibrational quantum number, *k*_B_ is the Boltzmann constant, and *T* is the sample temperature. The parameters used in the photoluminescence simulations were selected based on correlation with the experimental spectra and physical constraints of the Franck–Condon model. The electronic transition energy (*E*_00_ = *ℏ*ω_*ab*_) was estimated from the position of the emission maximum, while the line width parameter (*d*) was tuned to reproduce the observed spectral broadening, accounting for inhomogeneous disorder. The temperature was fixed at 300 K, consistent with the experimental conditions. The Huang–Rhys factors (*S*_*j*_) were treated as fitting parameters, constrained within physically reasonable values, to quantify the strength of electron–phonon coupling.

Equation 3 describes the corrected photoluminescence intensity and line shape considering the effect of self-absorption, i.e., the reabsorption of emitted photons in the spectral region where absorption and emission overlap:^36^

$$I\left(\lambda \right)=I_{0}\left(\lambda \right)\frac{1-e^{-A\left(\lambda \right)}}{A\left(\lambda \right)}$$ 3

where *I*_0_(λ) is the corrected emission intensity, *I*(λ) is the measured intensity, and *A*(λ) is the absorbance of the material.

## 3 Results and Discussion

The Cur–Zn, Cur–Ca, and Cur–Pb complexes exhibit dark-orange, red, and reddish-brown coloration, respectively. In contrast to free curcumin, which is readily soluble in ethanol, the synthesized complexes are insoluble in this solvent but display good solubility in polar aprotic solvents such as DMF and DMSO. Structural characterization was performed using Fourier transform infrared (FTIR) and Raman spectroscopies. Figure 2 presents the overlaid FTIR spectra of all samples, while the individual spectra are provided in Figures SI12–SI15 of the Supporting Information. The Cur–Zn and Cur–Pb complexes display closely similar spectral profiles that are clearly distinct from that of pristine curcumin. Conversely, the Cur–Ca complex does not exhibit well-defined curcumin-related features, which may be attributed to the high calcium acetate content and the relatively low complexation efficiency. The absorption band at 1272 cm^–1^, assigned to the phenolic C–O stretching vibration, is preserved in all complexes, indicating that the lateral aromatic rings are not involved in metal coordination. In contrast, the central C=O stretching mode at 1627 cm^–1^ undergoes a systematic shift toward lower wavenumbers upon complexation, consistent with coordination of the carbonyl group to the metal center. Additional downshifts are observed at 1506 cm^–1^, associated with aliphatic chain vibrations, and at 1425 cm^–1^, attributed to the enolic C–O stretch, further supporting metal–ligand interactions within the central β-diketone moiety. Moreover, the reduced intensity in the 3200–3500 cm^–1^ region, corresponding to O–H stretching vibrations, suggests the involvement of the enolic hydroxyl group in coordination. This assignment is further corroborated by the emergence of new low-frequency bands at 484 cm^–1^ for Cur–Zn and 522 cm^–1^ for Cur–Pb, which are characteristic of metal–oxygen stretching modes. Taken together, these spectroscopic features demonstrate that metal ions preferentially coordinate to the central β-diketone region of curcumin, consistent with a 2:1 curcumin-to-metal stoichiometry. This coordination behavior is consistent with recent vibrational studies of curcumin–metal complexes, reporting similar frequency shifts and coordination patterns involving the enolic and carbonyl groups.^24,25^ Following the work of Krishnankutty et al.,^37^ who synthesized curcumin complexes with different metals such as Cd­(II), Hg­(II), Pb­(II), Sn­(II), and Ca­(II) to evaluate their antimicrobial properties, a similar synthetic route was employed in our study. Complexes with comparable colors were obtained, with the Pb complex exhibiting a brownish-red color and the Ca complex appearing red. Structural characterization by mass spectrometry, FTIR, and NMR indicated an ML_2_ stoichiometry. FTIR analysis reveals that curcumin exhibits an absorption band at 1627 cm^–^, assigned to the intramolecularly hydrogen-bonded carbonyl group. In the complexes, this band is shifted to lower wavenumbers, being observed at 1615 cm^–1^ for Cur–Pb, 1621 cm^–1^ for Cur–Zn, and 1607 cm^–1^ for Cur–Ca. This shift is due to the coordination of the carbonyl group to the metal center; in agreement with previous reports.^37–39^ Further evidence of carbonyl involvement is the appearance of two medium-intensity peaks in the 470 to 420 cm^–1^ region, characteristic of M–O bonds, for the Cur–Pb complex around 470 cm^–1^, Cur–Zn at 472 cm^–1^, and Cur–Ca at 485 cm^–1^.

**Figure 2:**
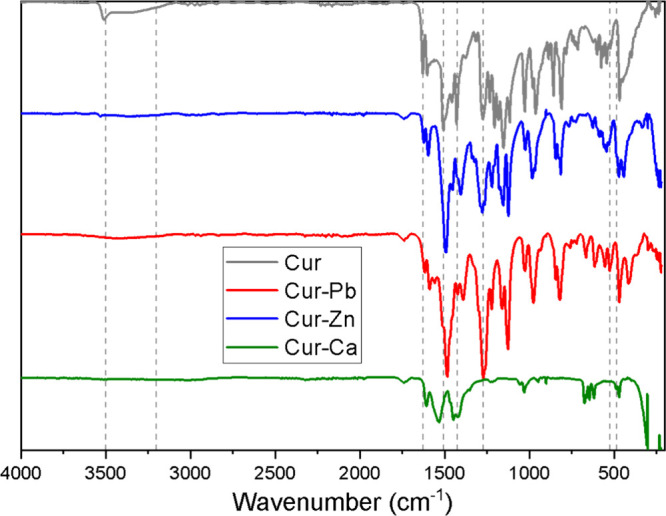
FTIR spectra of powdered Cur, Cur–Pb, Cur–Zn, and Cur–Ca samples. Each spectrum was normalized to its maximum intensity and vertically offset to facilitate comparison.

Raman spectroscopy was performed on the powdered samples immediately after synthesis, without dilution, to avoid spectral interference from DMSO, whose Raman bands overlap with those of curcumin and its metal complexes. Figure 3 presents the Raman spectra of pristine curcumin (Cur) and the corresponding metal complexes. Noticeable variations in peak positions, intensities, and line shapes are observed upon complexation, indicating structural modifications of the curcumin framework and electronic interactions with the metal centers. The individual Raman spectra of the complexes are provided in Figures SI7–SI10 of the Supporting Information.

**Figure 3:**
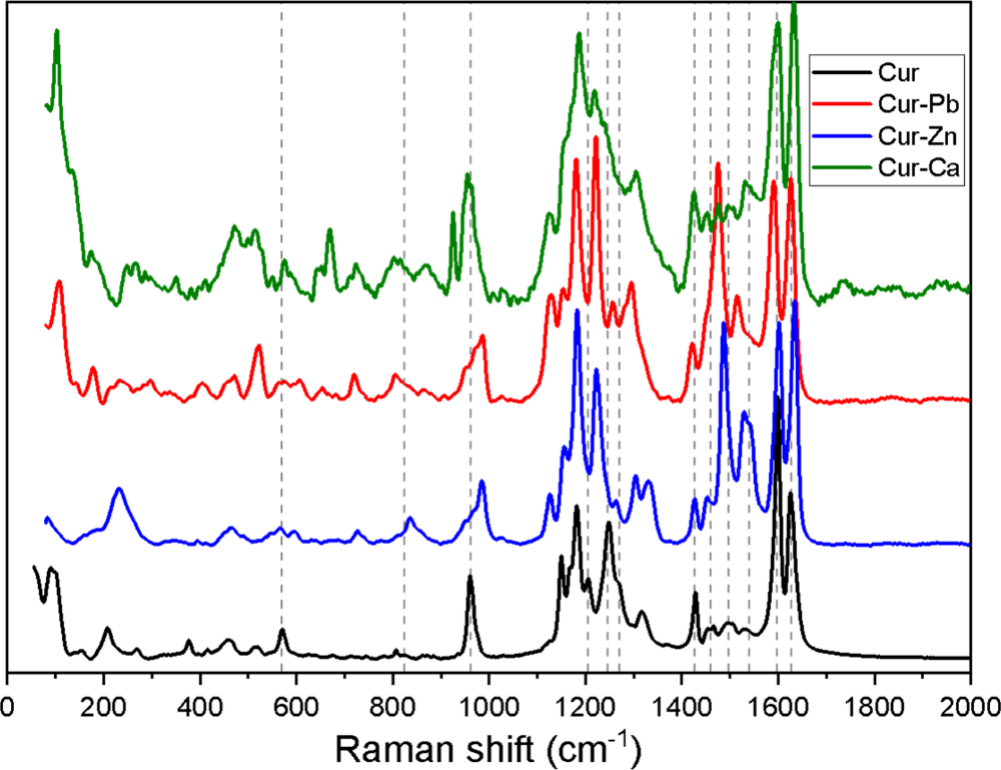
Raman spectra of powdered samples of Cur, Cur–Pb, Cur–Zn, and Cur–Ca. Each spectrum was normalized to its maximum intensity and vertically offset for clarity and ease of comparison.

From the deconvolution of the Raman spectrum (see Figure SI11 in the Supporting Information), Table 2 was constructed, correlating wavenumbers with the corresponding vibrational modes of curcumin.^47–54^ This table allows identification of changes in the complexes, such as shifts or suppression of vibrational modes, enabling a preliminary assessment of their structural modifications. Comparison of the Raman spectra of Cur–Zn, Cur–Pb, and Cur–Ca with that of pure curcumin reveals characteristic shifts indicative of metal–ligand interactions. In Cur–Zn, a slight red shift is observed in the aromatic ring modes (1496–1460 and 572 cm^–1^), accompanied by a blue shift in the enolic C–O stretching vibration (1207 cm^–1^). The decreased intensity of the phenolic C–OH peak (1247 cm^–1^) and shifts in the central C=O vibrations (965 and 1628 cm^–1^) suggest coordination via the enolic oxygen, while the central C=O remains largely intact, consistent with a stoichiometry of two curcumin molecules per Zn atom.

**Table 2:** Raman Shift Values for the Curcumin Sample (Cur) and Their Corresponding Vibrational Modes, Obtained from the Deconvolution of the Raman Spectrum Shown in Figure SI3 of the Supporting Information

Raman shift (cm^–1^)	functional group
570.0 ± 0.1	R–OCH_3_ methoxy group
824.0 ± 0.1	CH out-of-plane bending of aromatic CCH and skeletal CCH
962.0 ± 0.2	in-plane bending of CCH/C–OH/C–O–C/C=O
1150.0 ± 0.2	C–H/in-plane bending aromatic CCH and skeletal CCH
1180.1 ± 0.1	C–O/CH_3_/C–O–C stretching
1206.3 ± 0.3	enol C–O stretching
1247.2 ± 0.2	C–OH stretching enol
1271.1 ± 0.6	CH in-plane bending of C=CH, aromatic C–O stretching
1318.0 ± 0.4	C=CH stretching inter-ring
1427.3 ± 0.1	C–O stretching phenol, in-plane bending of aromatic CCC/CCH
1461 ± 1	CH_3_ stretching
1497 ± 1	CH_3_ stretching
1541 ± 6	C=O stretching, CCC/CC=O in-plane bending
1598.4 ± 0.1	inter-ring C=C bending
1628.3 ± 0.1	inter-ring C=C bending, C=O stretching

In Cur–Pb, similar trends are observed, including reduced C–OH intensity and a blue shift at 965 cm^–1^, whereas the aromatic ring vibrations shift to lower energies (1427, 1460, and 1598 cm^–1^), indicating increased vibrational flexibility. For Cur–Ca, interactions with the central β-diketone region are evident (blue shift at 965 cm^–1^ and red shift at 1247 cm^–1^), although peaks from residual calcium acetate suggest lower complexation efficiency. The presence of both intact and modified curcumin peaks indicates partial interaction with the metal center.

The Raman spectra of free curcumin and its complexes (Figure 3) show that the band at ∼1625 cm^–1^, associated with the conjugated β-diketone system and resulting from the coupled ν­(C=O) and ν­(C=C) stretching modes, undergoes splitting and a change in the intensities of these two peaks after complexation, indicating coordination through the β-diketone portion. Changes in intensity and broadening of the peaks are also observed in the region associated with the enol group (1300–1200 cm^–1^), suggesting the formation of a bond between the enolic oxygen and the metal, which supports the complex formation. Another piece of evidence for complex formation is the appearance of new bands in the low-frequency region (600–300 cm^–1^), which are absent in the Raman spectrum of free curcumin. Bands in this region can be attributed to ν­(M–O) stretching vibrations and δ­(M–O–C) bending vibrations.^40^ The similarity of the changes in the FTIR and Raman spectra observed for the Cur–Ca, Cur–Zn, and Cur–Pb complexes, as well as the observed colors of the complexes, corroborates the formation of complexes with ML_2_ stoichiometry, as previously described in the literature.^37–39^

Figure 4 shows normalized absorption and emission spectra of curcumin (Cur) and its metal complexes (Cur–Pb, Cur–Zn, and Cur–Ca) in DMSO solution. It should be noted that the solubility of curcumin in organic solvents such as DMSO, ethanol, and DMF is significantly higher than in aqueous media.^1,41^ In contrast, the metal complexes exhibit limited solubility in most organic solvents.^7^ The absorption spectrum of curcumin displays a broad band in the 300–500 nm region, with a maximum at approximately 424 nm, associated with the π → π* transition in the presence of a polar solvent such as DMSO.^1,42–44^ This band may overlap with the *n* – π* transition, as solvent–solute interactions promote band superposition, with the π → π* transition occurring at lower energies.^3^ When comparing the spectra of the complexes with that of free curcumin, a red shift of approximately 2–8 nm is observed, with maxima in the range of 426–432 nm. This shift suggests coordination of the carbonyl groups to the metal center, since these groups are primarily responsible for changes in absorption maxima, as also evidenced in solvent-dependent studies that account for the tautomeric nature of curcumin.^1,22,41^ Such spectral shifts have been widely associated with ligand-to-metal or intraligand charge-transfer contributions in recently reported curcumin-based complexes.^26^ In addition, distinct shoulders are observed in the complexes at 408–412 and 448–452 nm. These features can be attributed to charge-transfer interactions between curcumin and metal ions, as reflected by the enhanced absorption intensity relative to free curcumin in these regions.^22,45^ Absorption bands between 500–600 nm are typically associated with d–d transitions in Zn­(II) complexes.^46^

**Figure 4:**
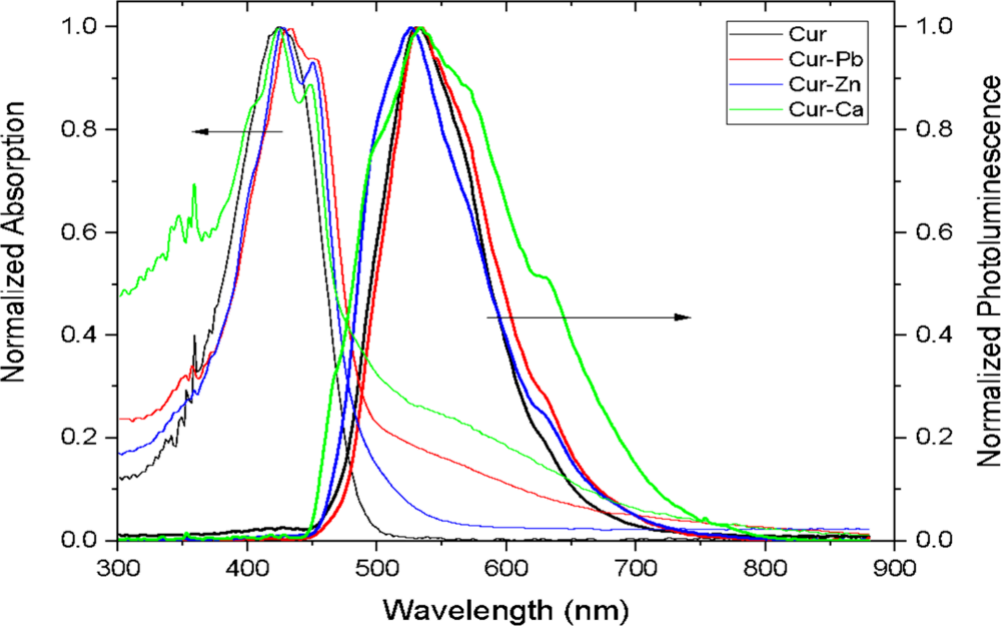
Normalized absorption and emission spectra of curcumin (Cur) and its metal complexes (Cur–Pb, Cur–Zn, and Cur–Ca) in DMSO solution.

The complexes were obtained in varying yields 85% for Cur–Zn, 75% for Cur–Pb, and 14% for Cur–Ca. The complexes formed with Zn and Pb show higher yields because coordination with the β-diketonate moiety of curcumin is favored, leading to the formation of more stable complexes. The reaction of curcumin with Ca, on the other hand, shows a lower yield, possibly due to the more labile nature of this metal. It is worth noting that Ca^2+^ prefers higher coordination numbers of ligands, because it is better stabilized by ligands that provide multiple coordination sites. In this system, the curcumin acts as a bidentate ligand, it may not fully satisfy the coordination sphere of Ca^2+^. It is worth noting that Ca forms more stable complexes with ligands that can perform a greater number of coordination sites. Therefore, the formation of soluble species in the reaction medium may contribute to the reduced yield. The stability of compounds can be evaluated from the absorption and emission spectra of the complexes, shown in Figure 4. It can be observed that the spectra exhibit distinct behaviors. Curcumin displays its characteristic band at 425 nm assigned to the π–π* transitions of the conjugated β-diketonate system. In the Zn and Pb complexes, a red shift is observed in this band, as shown in Table 3. This suggests significant electronic interaction between the metal and the curcumin ligand. A shift in the emission spectrum of these two complexes is also observed, indicating an increase in structural rigidity and consequently stability. In contrast, Cur–Ca shows virtually no shifts in absorption and emission wavelengths relative to the spectra of free curcumin, suggesting a weaker interaction and lower complexation efficiency compared to Zn and Pb. Analyzing the Stokes shifts (Δν̅) for the complexes, it is observed that Cur–Zn exhibits the lowest Δν̅ value, indicating less structural relaxation and consequently greater stiffness in the excited state. A similar behavior is observed for Cur–Pb. The Cur–Ca complex presents the highest Stokes shift, similar to free curcumin, indicating flexibility and weaker coordination in solution.

**Table 3:** Maximum Absorption and Emission of Free Curcumin and Its Complexes, and Stokes Shifts (Δν̅)

sample	λ_abs_ (nm)	ν_abs_ (cm^–^^1^)	λ_em_ (nm)	ν_em_ (cm^–^^1^)	Δν̅ (cm^–^^1^)
Cur	425	23,529	531	18,832	4697
Cur–Ca	425	23,529	533	18,762	4767
Cur–Zn	429	23,310	526	19,011	4299
Cur–Pb	434	23,041	533	18,762	4279

All PL spectra were corrected using eq 3, and the comparisons with the experimental spectra are provided in the Supporting Information (Figures SI3–SI6). It is well established that the solvent is the most influential factor governing the position of the luminescence maximum in curcumin, which can range from 446 nm in cyclohexane to 560 nm in methanol.^43^ The emission spectrum (Figure 4) exhibits a broad band between 450 and 800 nm, with a maximum at 530 nm for free curcumin, consistent with the expected range for polar solvents such as DMSO.^1,43–46^ The metal complexes display emission maxima in the 525–534 nm range, presenting a shift of less than 5 nm relative to free curcumin (Figure 4). These small variations can be attributed to the influence of the metal on the central β-diketone moiety of curcumin. In the first singlet excited state (*S*_1_), curcumin undergoes substantial intramolecular charge transfer from the terminal aromatic rings toward the central carbonyl/enol groups (Figure 1; see also ref 36). Coordination to the metal may alter the local conformation and modulate out-of-plane vibrational modes, thereby affecting the emission profile.

The photoluminescence line shape analysis provides deeper insight into the effects of metal coordination on the electronic and vibronic structure of curcumin. Recent studies have highlighted that vibronic coupling and electron–phonon interactions play a central role in defining emission line shapes in metal–organic systems, particularly when coordination alters molecular rigidity and electronic delocalization.^25^ Figure 5 presents the comparison between the experimental spectra and the simulated curves, while the corresponding fitting parameters are summarized in Table 4. The simulations were performed using eqs 1 and 2, considering the electronic gap energy (*E*_00_) associated with the π–π* transition, the line width parameter (*d*), the temperature, and the Huang–Rhys factors (*S_j_*), which quantify the electron–vibrational coupling strength and vibrational energy (*E*_0*j*_) based on the Raman or FTIR data. The electronic gap energy *E*_00_ shows a clear dependence on metal coordination. While pristine curcumin and the Cur–Ca complex exhibit the same *E*_00_ value (514 nm), a noticeable blue shift is observed for Cur–Pb (500 nm) and Cur–Zn (492 nm). This trend suggests that heavier or more strongly interacting metal centers induce a stabilization of the ground state or a modification of the conjugation length, leading to an increased effective bandgap. Such behavior is consistent with metal–ligand interactions affecting the π-electron delocalization of the curcumin backbone.

**Figure 5:**
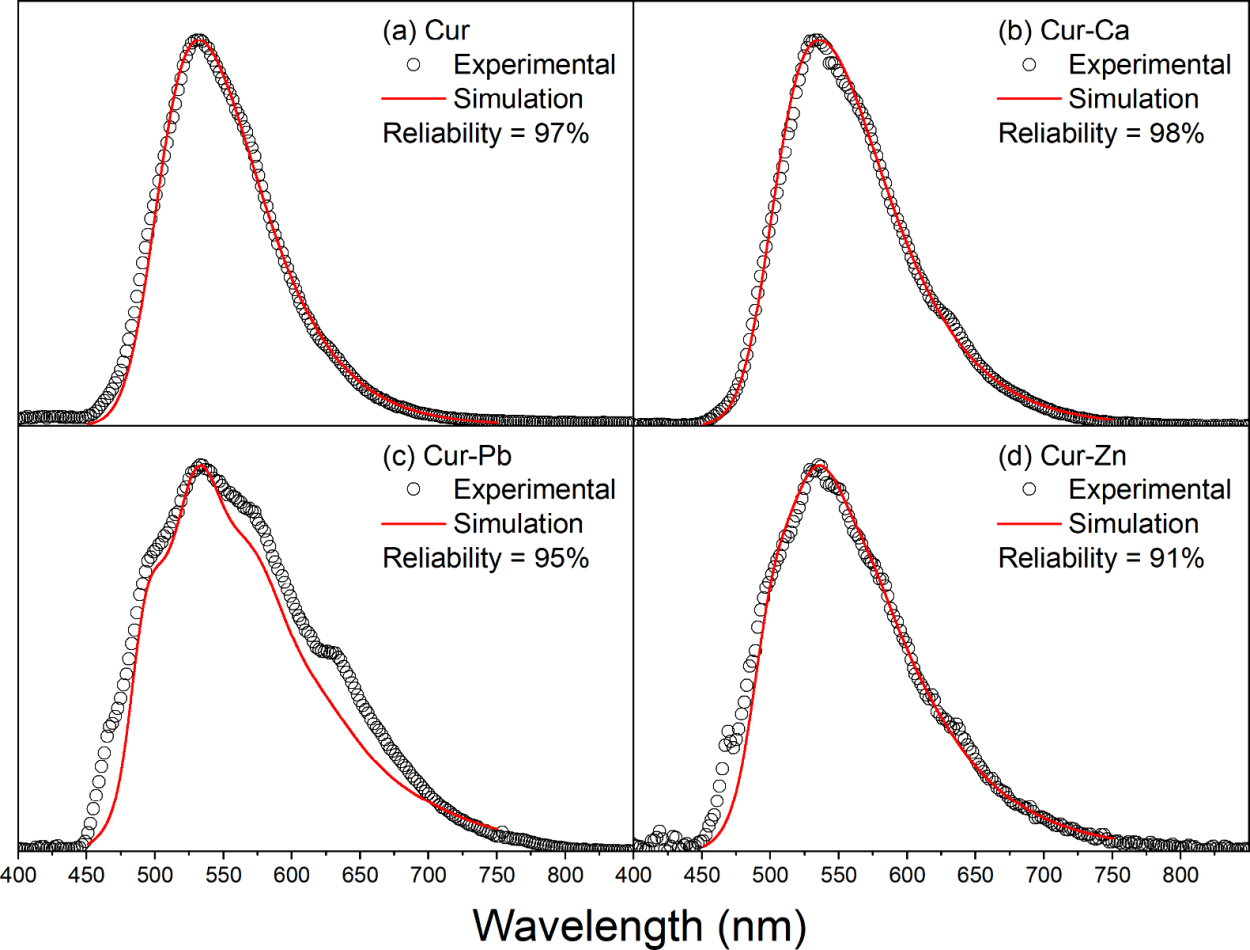
Simulation of photoluminescence spectrum of (a) Cur, (b) Cur–Ca, (c) Cur–Pb, and (d) Cur–ZN samples.

**Table 4:** Parameters Adjusted in the Simulations of the Photoluminescence Spectrum in Figure 5 of (a) Cur, (b) Cur–Ca, (c) Cur–Pb, and (d) Cur–Zn Samples

parameters	Cur	Cur–Ca	Cur–Pb	Cur–Zn
*E*_00_ (nm)	514	514	500	492
*d* (cm^–1^)	700	700	550	400
*T* (K)	300	300	300	300
*E*_01_ (cm^–1^)	570	570	570	570
*E*_02_ (cm^–1^)	1206	1206	1206	1206
*E*_03_ (cm^–1^)	1598	1598	1598	1598
*S*_1_	0.50	0.50	0.45	0.60
*S*_2_	0.55	0.60	1.00	0.65
*S*_3_	0.10	0.17	0.23	0.70
reliability	97%	98%	95%	91%

In parallel, the line width parameter *d* decreases systematically from Cur (700 cm^–1^) to Cur–Zn (400 cm^–1^). This reduction indicates a progressive decrease in energetic disorder and inhomogeneous broadening upon complex formation, particularly for the Zn-coordinated system. The narrower line width in Cur–Zn suggests a more rigid and structurally homogeneous emissive environment, likely arising from stronger coordination and reduced conformational freedom of the curcumin molecule.

The selection of the vibrational modes included in the simulations was guided by the experimental Raman and FTIR spectra. Three representative modes (≈570, 1206, and 1598 cm^–1^) were chosen due to their high intensity and clear assignment to distinguish structural units of the curcumin molecule, namely, the methoxy groups, the β-diketone moiety, and the aromatic C=C framework. These modes are expected to contribute most significantly to the vibronic progression in the emission spectra. It is worth noting that the use of a reduced number of effective vibrational modes is a common approach in vibronic modeling, allowing the main spectral features to be captured without introducing unnecessary fitting parameters. This approximation is particularly suitable for complex molecular systems, where a limited set of dominant modes governs the electron–vibrational coupling. The vibronic structure of the PL spectra was modeled by considering three effective vibrational modes identified from FTIR and Raman measurements: 570 cm^–1^ (methoxy R–OCH_3_ stretching, unit C), 1206 cm^–1^ (enolic C–O stretching, unit A), and 1598 cm^–1^ (inter-ring C=C bending, unit B). The corresponding Huang–Rhys factors reveal important trends. For pristine curcumin, relatively small *S* values indicate weak to moderate electron–phonon coupling. Upon metal coordination, however, a systematic increase in the Huang–Rhys factors are observed, particularly for modes *E*_02_ and *E*_03_. Notably, Cur–Pb and Cur–Zn exhibit significantly enhanced coupling to the higher-energy vibrational modes, with *S*_2_ and *S*_3_ reaching values as high as 1.00 and 0.70, respectively. This enhancement reflects stronger modulation of the excited electronic state by molecular vibrations, likely due to metal-induced changes in charge distribution and molecular rigidity. The pronounced increase in S_3_ for Cur–Zn suggests that coordination strongly affects the inter-ring C=C framework, reinforcing the role of metal binding in altering the vibronic landscape of curcumin. Overall, the high reliability values (above 90% for all samples) confirm the robustness of the simulation model and validate the assignment of the vibrational modes. The combined evolution of *E*_00_, line width, and Huang–Rhys factors demonstrates that metal coordination not only modifies the electronic structure of curcumin but also reshapes its electron–vibrational coupling, leading to distinct PL line shapes for each complex.

The magnetic field effects on pristine and metal-complexed curcumin were investigated using emission ellipsometry combined with Fourier-series deconvolution and the nine-point technique,^55^ both in the absence and in the presence of an external magnetic field. For each sample, measurements were performed under both conditions, as described in Section 2. This approach provides detailed insight into emission anisotropy, including the polarization state of the emitted light, and enables the identification of magnetic-field-induced contributions in curcumin-based systems. Figure 6 presents the results for the ellipsometric parameters *p*, *r*, and *g*, with the corresponding data provided in Figures SI16–SI41 and Tables SI1–SI4 of the Supporting Information. The parameter *S*_1_/*S*_0_ is directly associated with the linearly polarized component of the emission, whereas *p* represents the overall degree of polarization. A comparison between measurements performed in the absence and presence of the magnetic field reveals systematic variations in these parameters, indicating that the external field influences the polarization properties of the emitted light. In general, the metal–curcumin complexes exhibit more pronounced magnetic-field-induced changes than pristine curcumin, suggesting that metal coordination enhances magneto-optical effects. This behavior can be attributed to modifications in the excited-state electronic structure and radiative relaxation pathways arising from the interaction between the magnetic field and the metal centers, leading to changes in emission anisotropy.

**Figure 6:**
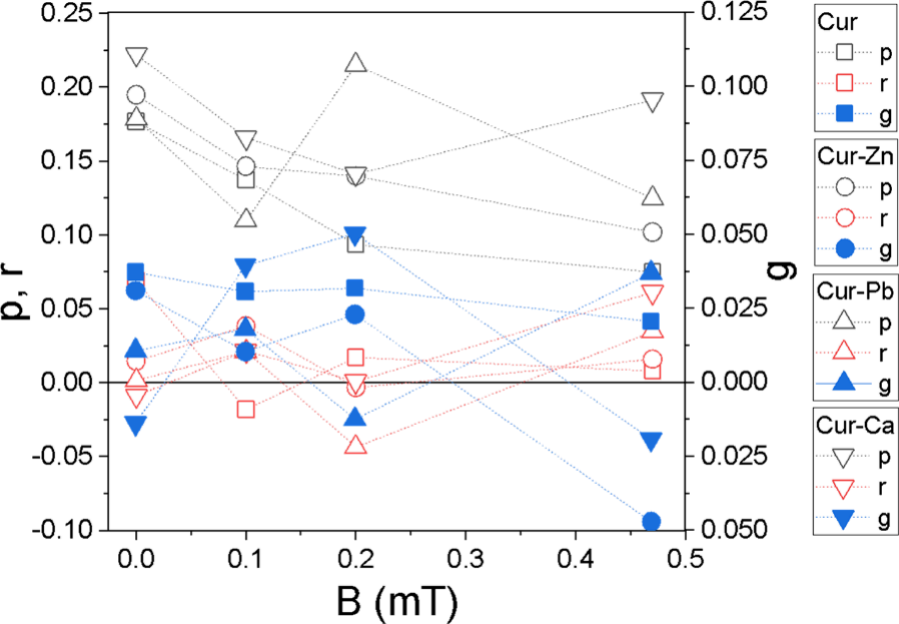
Magnetic field dependence of the ellipsometry parameters and *p* (black), *r* (red), and *g* (blue) for pristine curcumin (Cur, open squares) and the metal–curcumin complexes: curcumin–zinc (Cur–Zn, open circles), curcumin–lead (Cur–Pb, up triangles) and, curcumin–calcium (Cur–Ca, down triangles).

The remaining ellipsometry parameters, *S*_2_/*S*_0_ and *S*_3_/*S*_0_, as well as the derived parameters *r* and *g*, are shown in the Supporting Information (Figure SI17). For smaller magnetic field values (<0.47 mT) the asymmetry factor *g* remains close to zero, indicating no difference between right- and left-circularly polarized emissions. This result rules out the occurrence of circular dichroism in pristine or metal-complex curcumin under the experimental conditions employed. Likewise, the polarization factor *p* remains essentially constant with increasing magnetic field, as does the anisotropy factor *r*, which remains close to 5%. These findings are consistent with the isotropic nature of the sample in solution, where no preferential emission direction is expected. In contrast, the zinc– and calcium-curcumin complexes exhibit a clear magnetic field dependence at 0.47 mT. As shown in Figure 6, the *g* parameter displays a gradual change with increasing magnetic field intensity, indicating a tendency toward inversion the symmetry of the molecule induced via magnetic-field-induced circular dichroism.

## 4 Conclusions

The synthesis and comprehensive spectroscopic investigation of curcumin complexes with Zn­(II), Pb­(II), and Ca­(II) demonstrate that metal coordination occurs preferentially through the enolized β-diketone moiety of the curcumin molecule. FTIR and Raman analyses consistently indicate the involvement of both carbonyl and enolic hydroxyl groups in metal binding, while preserving the structural integrity of the terminal aromatic rings. These findings support a coordination model dominated by interactions within the central conjugated framework of curcumin. UV–vis absorption and photoluminescence measurements reveal systematic spectral shifts upon complexation, reflecting metal-induced perturbations of the electronic structure and the emergence of charge-transfer contributions. Vibronic line shape simulations show that metal coordination leads to significant changes in the electronic gap, electron–phonon coupling, and inhomogeneous broadening. In particular, the Zn­(II) complex exhibits a marked reduction in spectral line width, consistent with enhanced molecular rigidity and reduced conformational disorder. Magnetic field effects were characterized using an ellipsometry-based approach to probe the tuning of emission, revealing that metal coordination significantly influences the polarization state of curcumin emission. Among the investigated systems, the Zn­(II) complex exhibits the most pronounced magnetic-field-dependent response, including a change in the dominant linear polarization component. Overall, this work demonstrates that metal coordination is an effective strategy for tuning the electronic and vibronic properties of curcumin under external magnetic fields. The combined spectroscopic and modeling approach provides new insights into structure–property relationships in curcumin-based metal complexes and underscores their potential for applications in photonics, sensing, and biomolecular systems.

## Supplementary Material



## Notes

Sample pictures; photoluminescent setup scheme image; sample holder images and schematics; photoluminescence, Raman, FTIR, and emission ellipsometry spectrum of each sample; Raman spectrum analysis in Figures SI0–SI40; and emission ellipsometry data in tables SI1–SI4 (PDF)
