# Supplementary material for: Tuning Photoluminescence and Emission Polarization in Metal–Curcumin Complexes via Magnetic Fields
Source: ACS Omega. 2026 Jun 30;11(27):40365–73. doi: 10.1021/acsomega.6c03000 (PMC13382756; doi:10.1021/acsomega.6c03000)
Supplement: Supplementary file 1 [file ao6c03000_si_001.pdf]

Support Information

Tuning Photoluminescence and Emission Polarization in Metal–Curcumin Complexes via Magnetic Fields

Pedro Henrique Dondori Zaramella[1], Bruno Souza Zanatta[1*], Giovani Bortolini de Oliveira[1], Fernando Henrique Cristovan[2], Tatiane Moraes Arantes[2], Welington de Oliveira Cruz,[3] Raigna Augusta da Silva[1], Alexandre Marletta[1], Erick Piovesan[1].

[1] Physics Institute, Federal University of Uberlandia, Uberlandia, 38400-902, Brazil

[2] Institute of Exact Sciences and Technology, Federal University of Jataí, Jataí, 75801-615, Brazil

[3] Chemistry Institute, Federal University of Uberlandia, Uberlandia, 38400-902, Brazil

*Corresponding author: brunosouzanatta@ufu.br

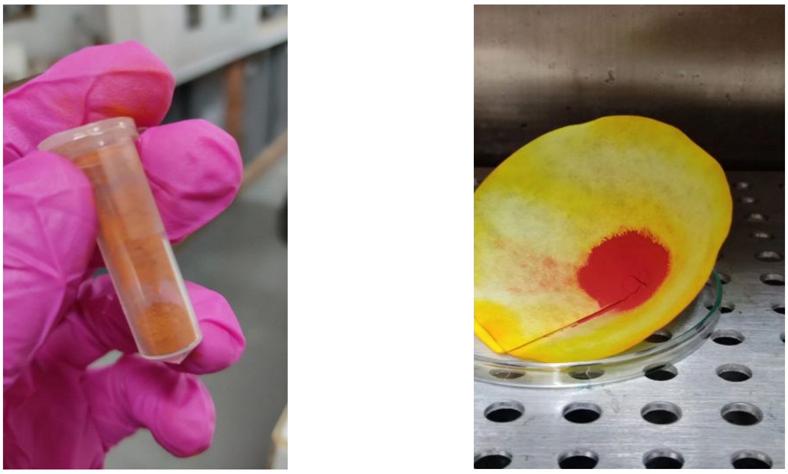

Cur-Zn e Cur-Pb

Figure SI0. Photographs showing the solid products obtained from the synthesis of the curcumin–zinc (Cur-Zn, left) and curcumin–lead (Cur-Pb, right) complexes.

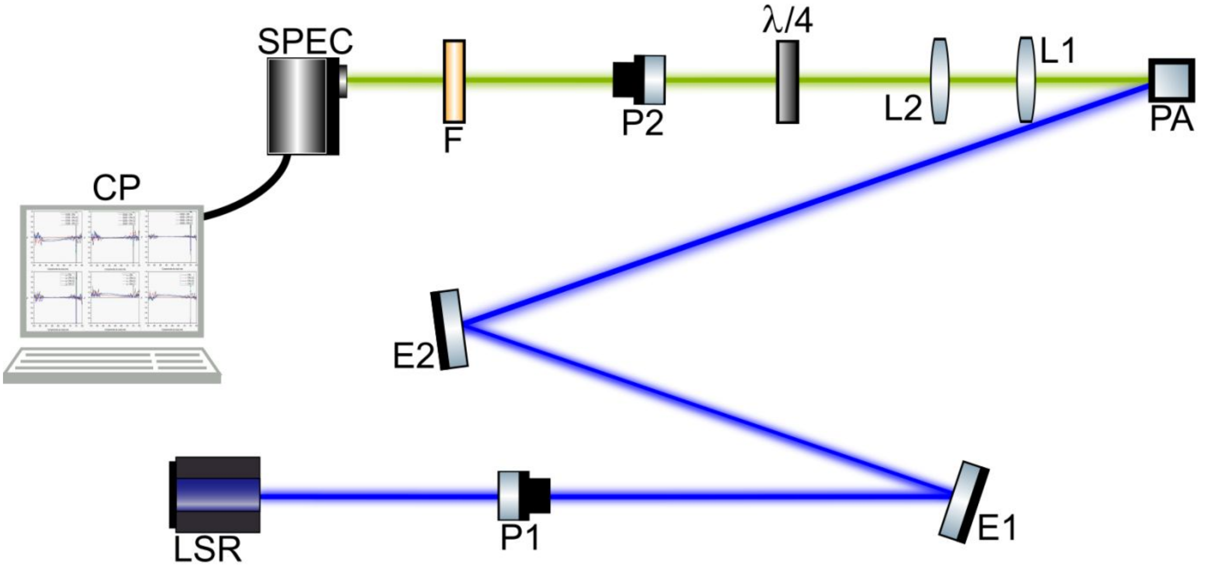

Figure SI1. Photoluminescence setup comprising a laser (LSR), mirrors (E1 and E2), a sample holder, two lenses (L1 and L2), an intensity filter (F), a spectrophotometer (SPEC), a computer (CP), two polarizers (P1 and P2), and a quarter-wave plate (λ/4).

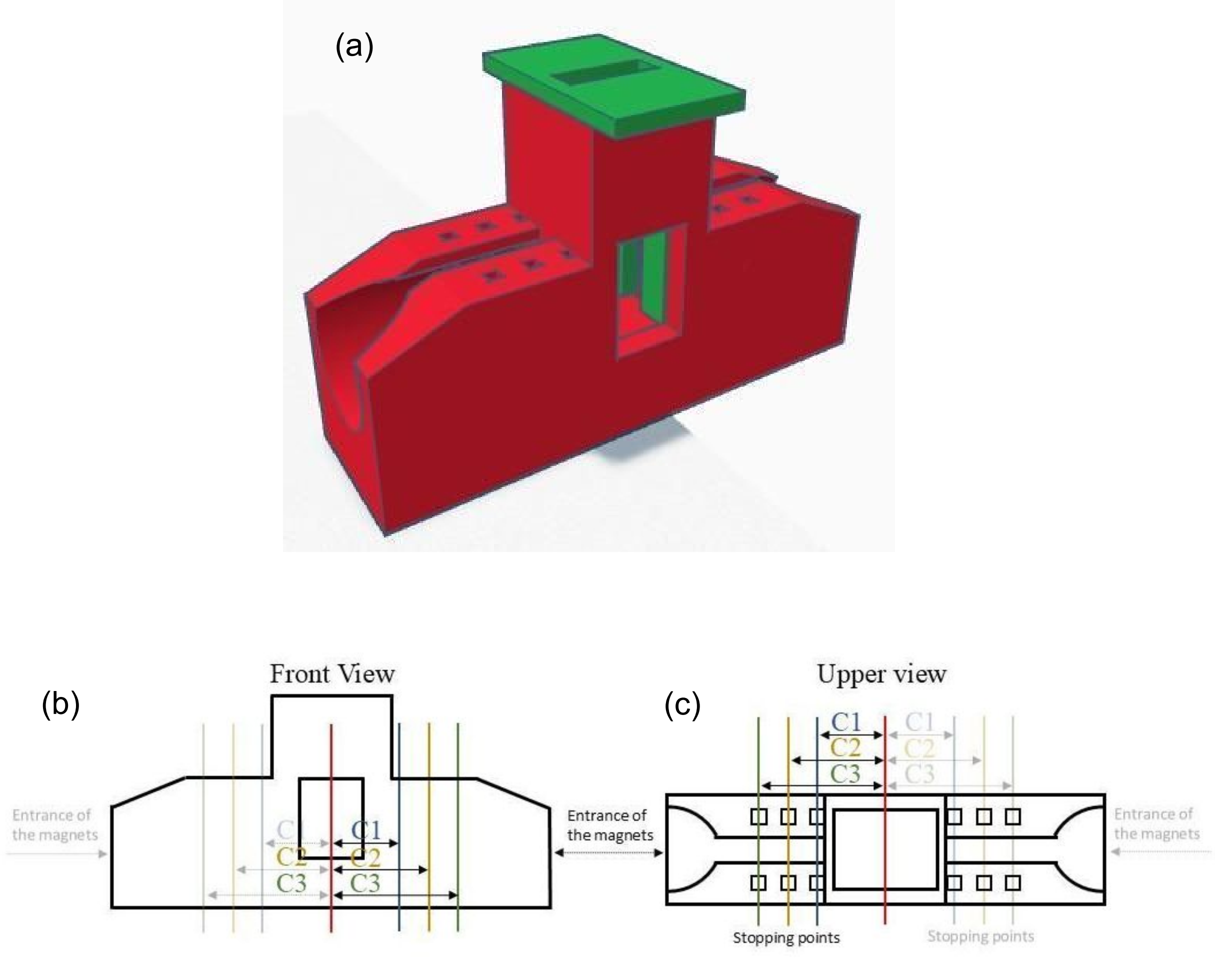

Figure SI2. (a) Sample holder used for magneto-optical measurements, with upper holes corresponding to the C1, C2, and C3 position locks. (b) Front view and (c) top view showing the C1–C3 positions.

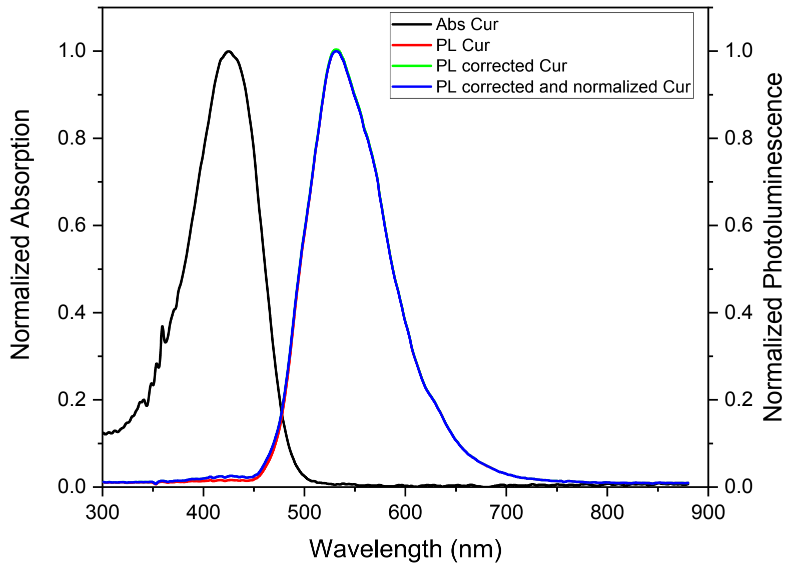

Figure SI3. Self-absorption correction of the Cur PL spectrum.

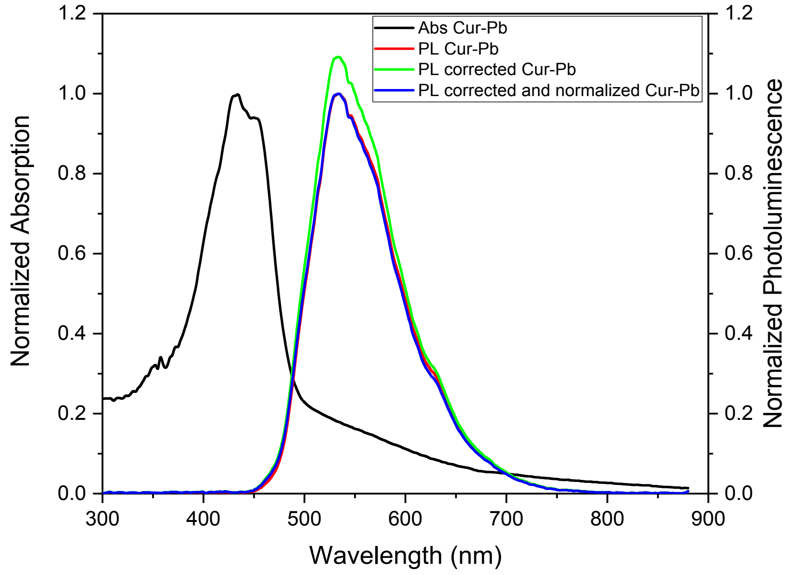

Figure SI4. Self-absorption correction of the Cur-Pb PL spectrum.

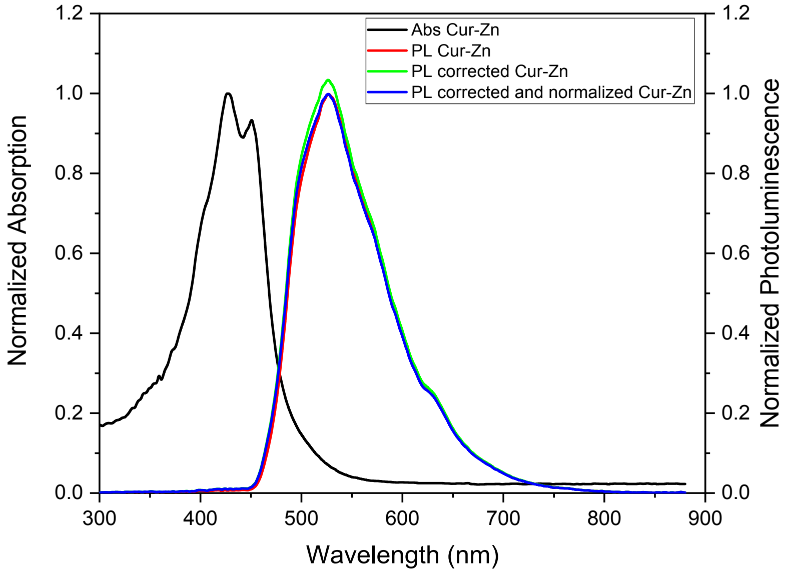

Figure SI5. Self-absorption correction of the Cur-Zn PL spectrum.

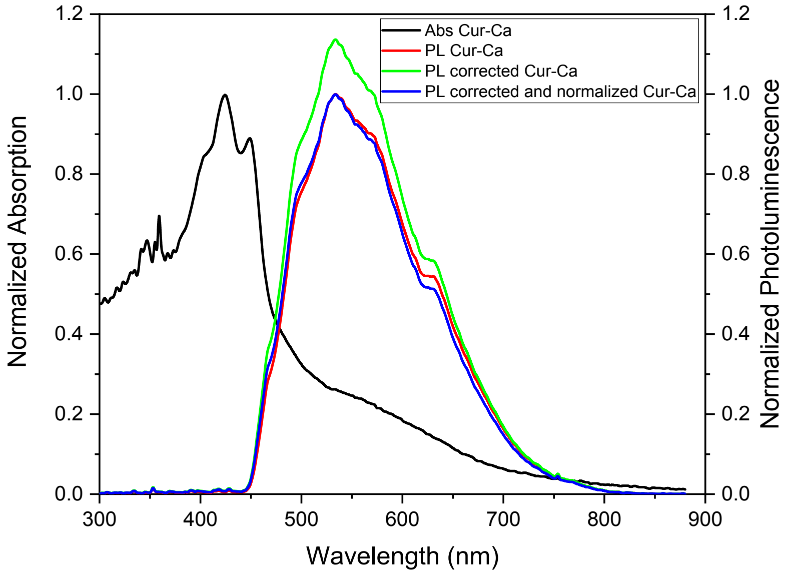

Figure SI6. Self-absorption correction of the Cur-Ca PL spectrum.

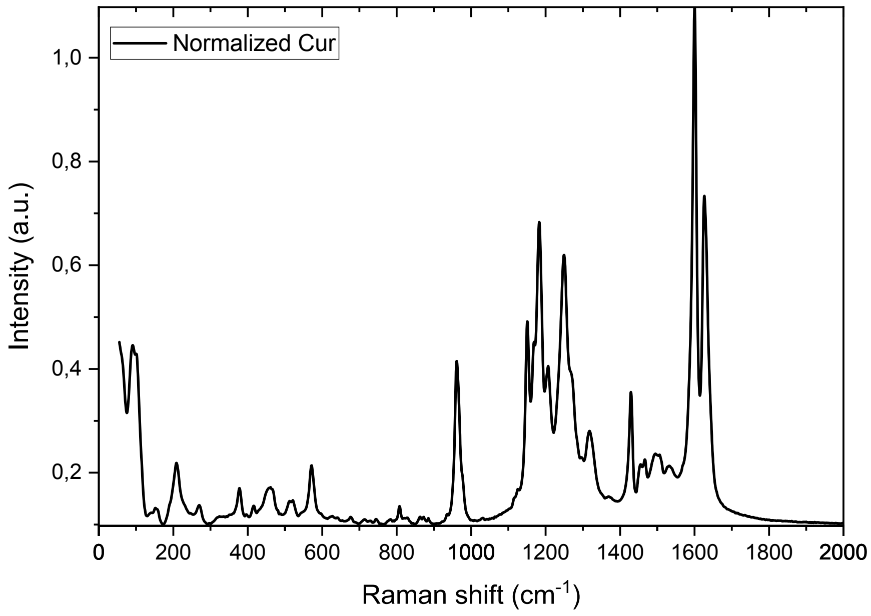

Figure SI7. Normalized Raman spectrum of Cur.

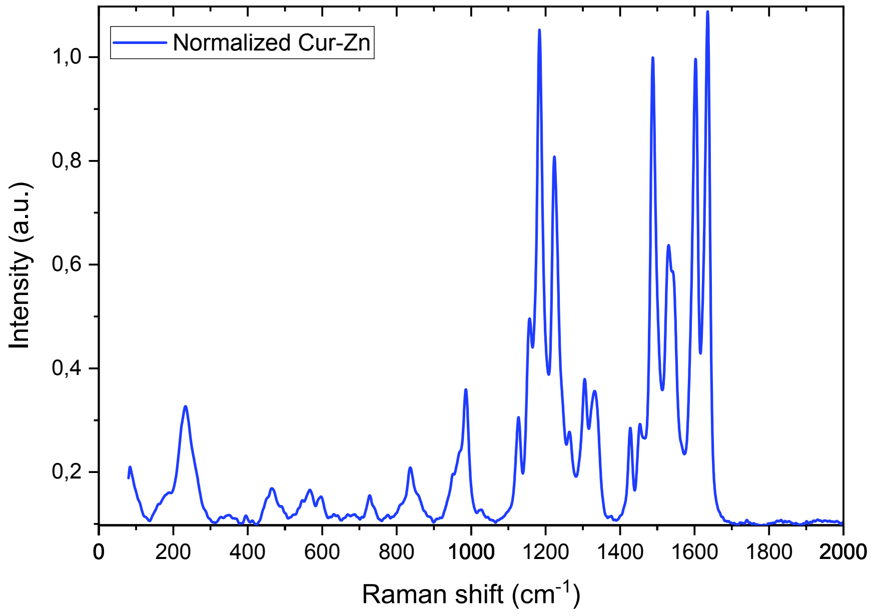

Figure SI8. Normalized Raman spectrum of Cur-Zn.

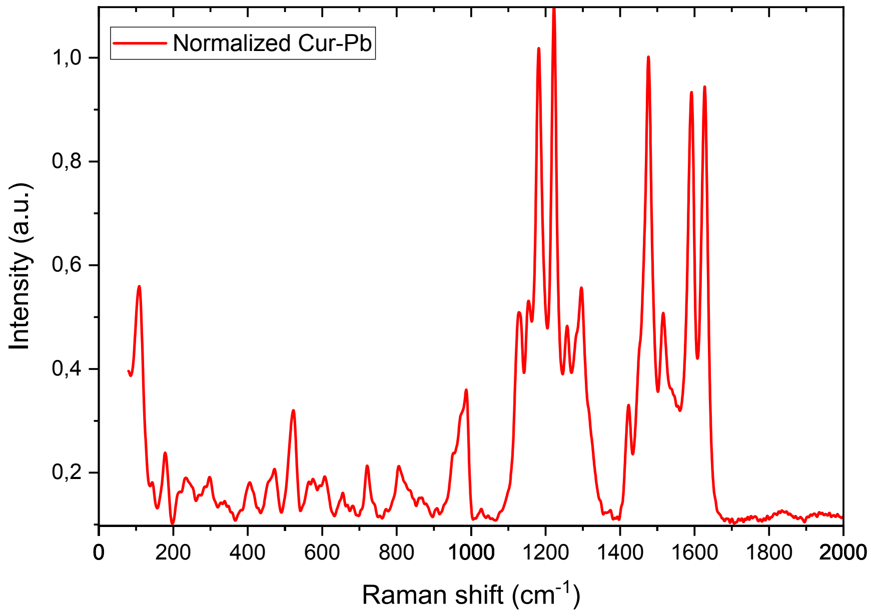

Figure SI9. Normalized Raman spectrum of Cur-Pb.

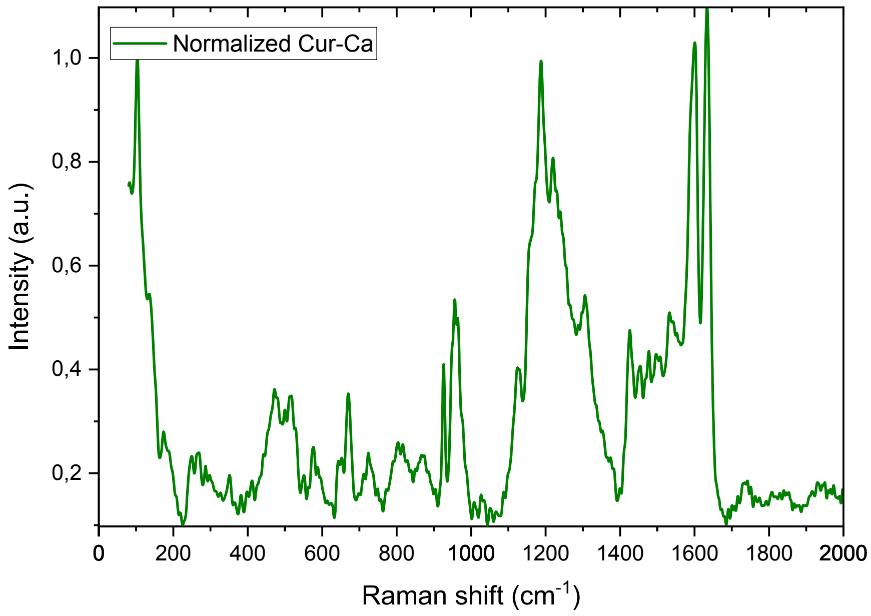

Figure SI10. Normalized Raman spectrum of Cur-Ca.

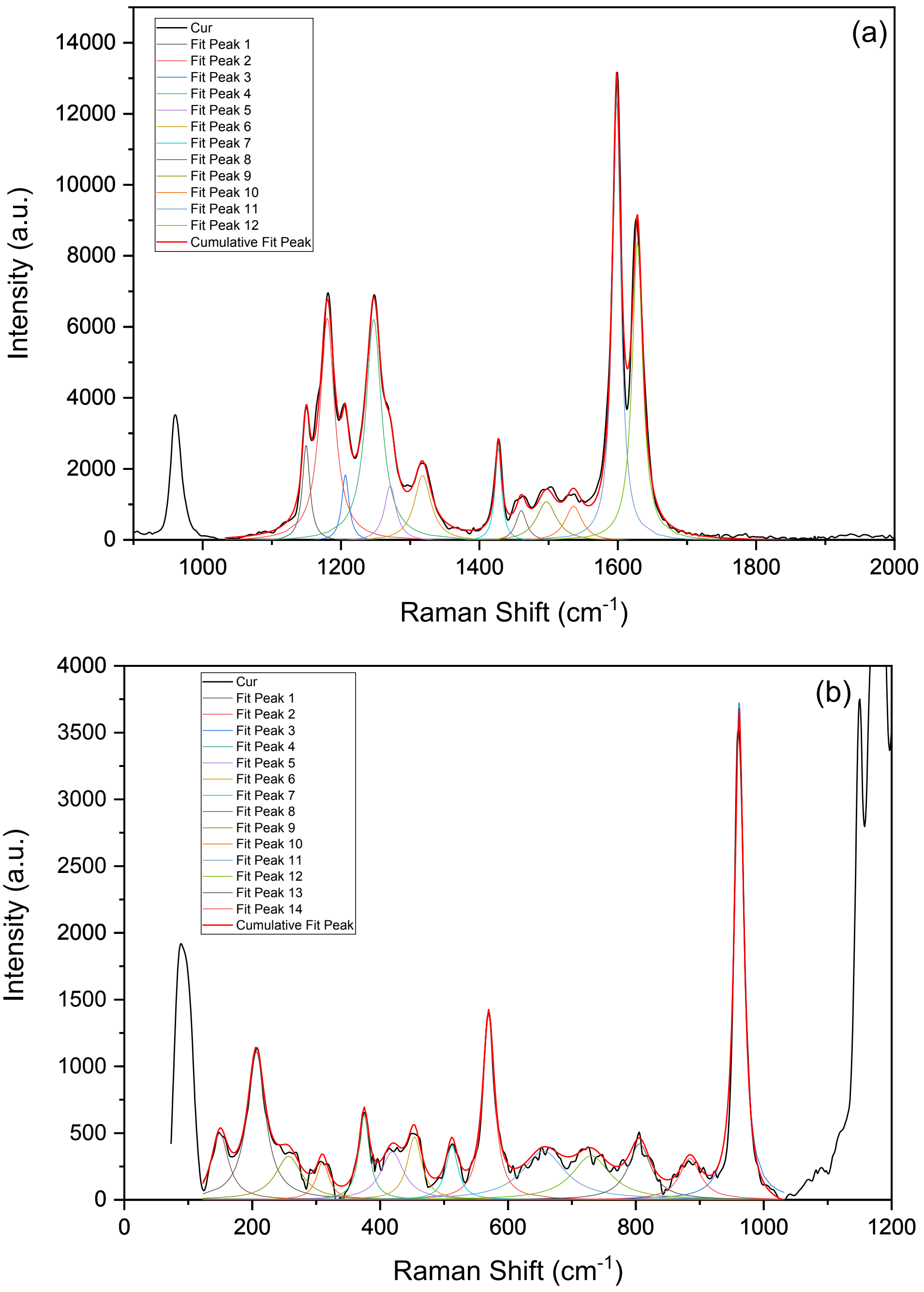

Figure SI11. Deconvolution of the Raman spectrum of Cur, from (a) 150 to 1020 cm^{-1}, and (b) 1020 to 1900 cm^{-1}.

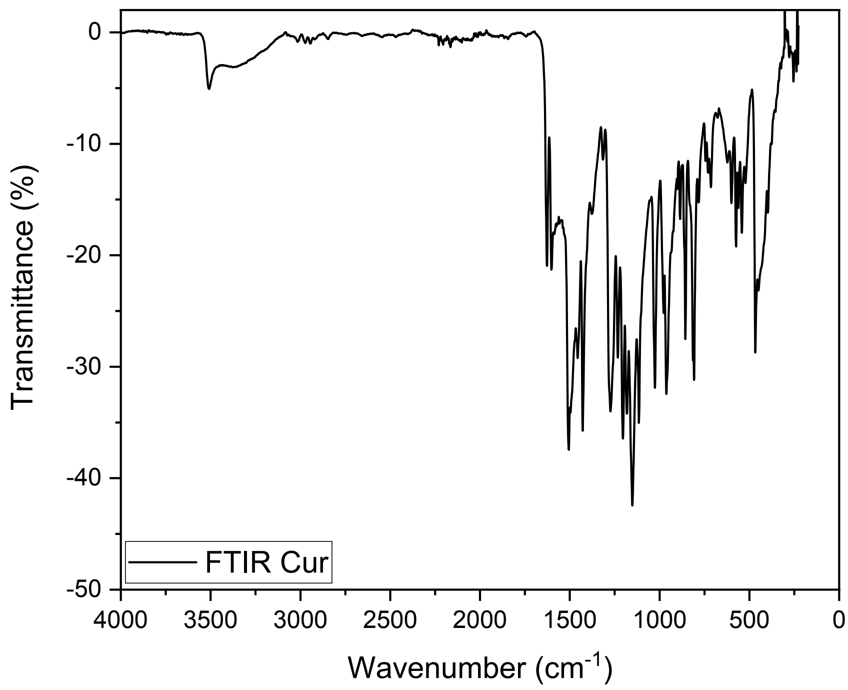

Figure SI12. FTIR spectrum of Cur.

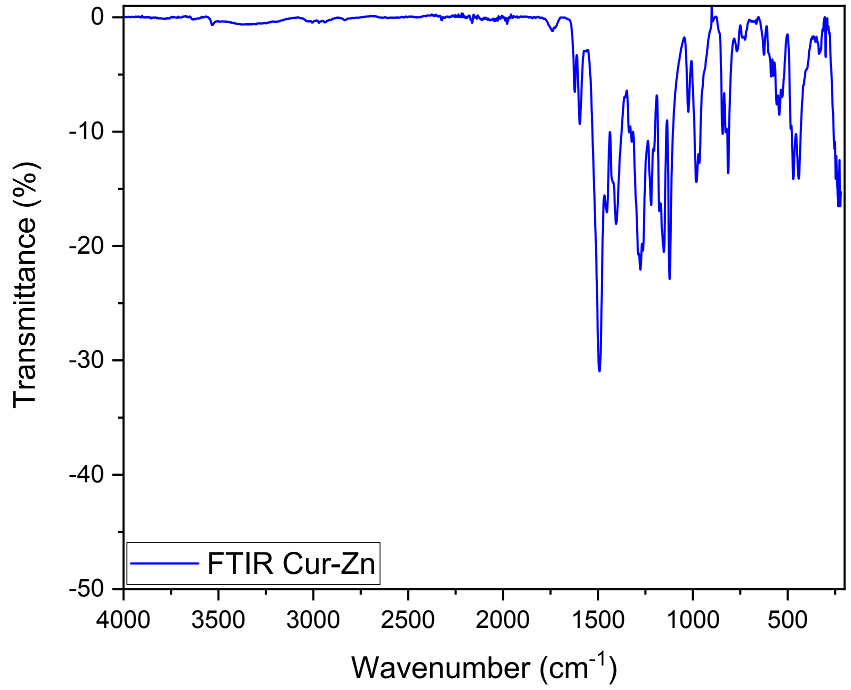

Figure SI13. FTIR spectrum of Cur-Zn.

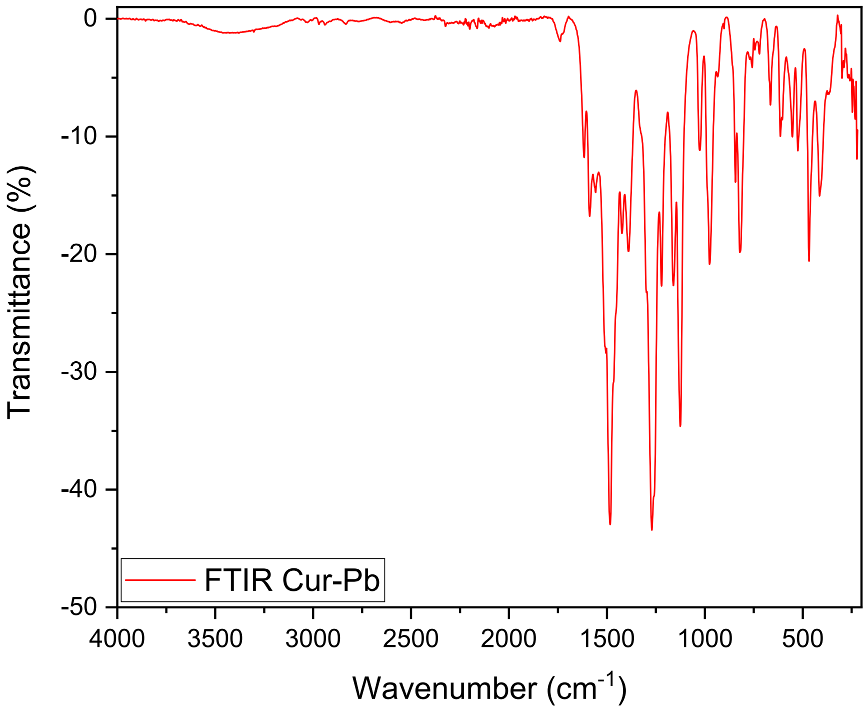

Figure SI14. FTIR spectrum of Cur-Pb.

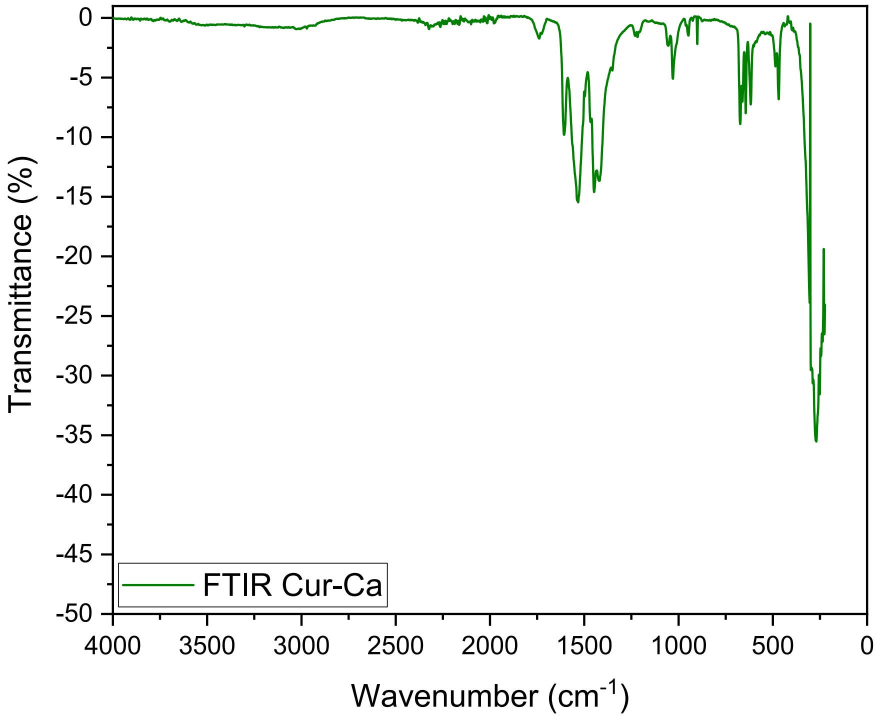

Figure SI15. FTIR spectrum of Cur-Ca.

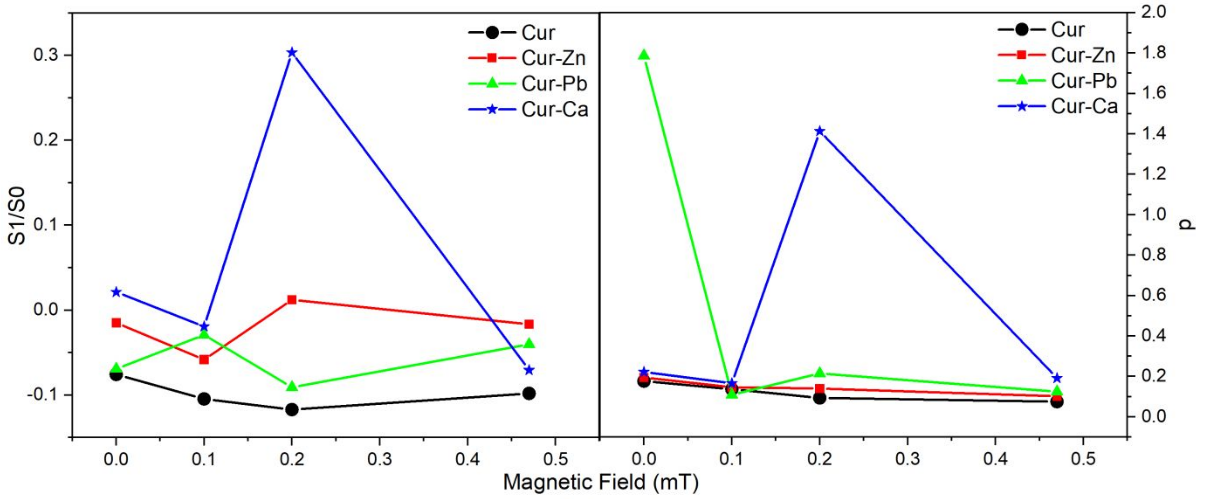

Figure SI16. S1/S0 ratio and degree of polarization (p) for Cur, Cur-Zn, Cur-Pb, and Cur-Ca.

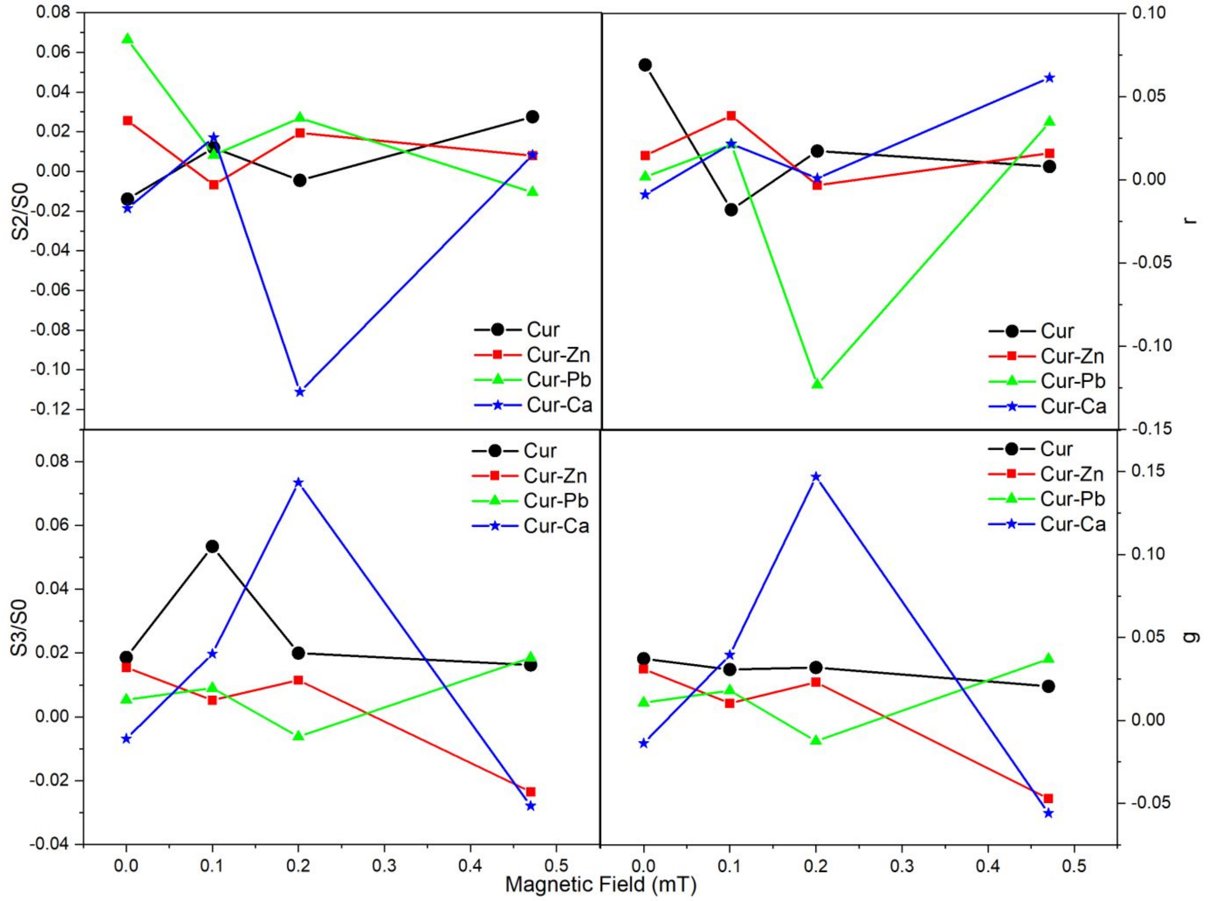

Figure SI17. S2/S0 and S3/S0 ratio, anisotropy (r) and asymmetry (g) factor for Cur, Cur-Zn, Cur-Pb, and Cur-Ca.

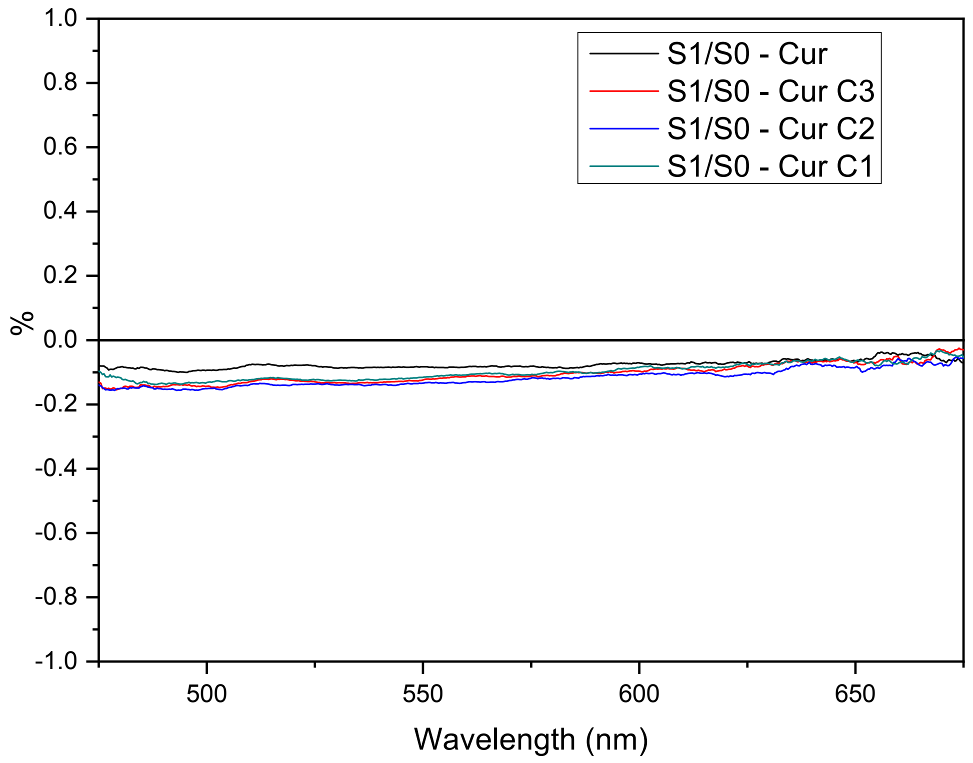

Figure SI18. S1/S0 ratio of Cur, with and without an applied magnetic field.

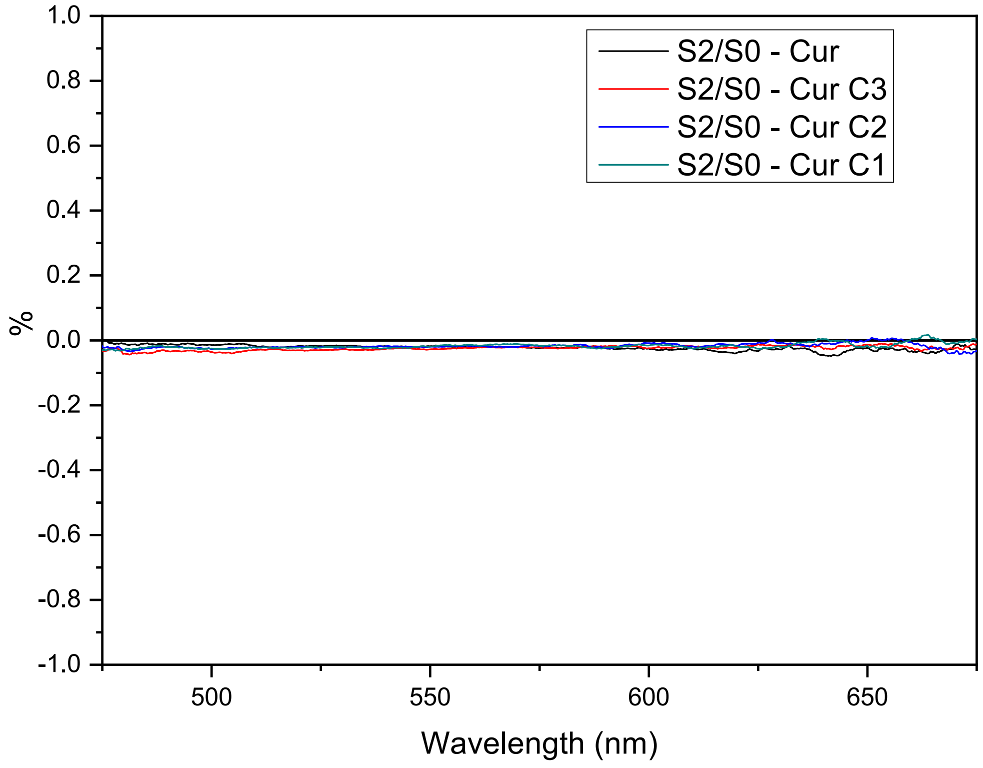

Figure SI19. S2/S0 ratio of Cur, with and without an applied magnetic field.

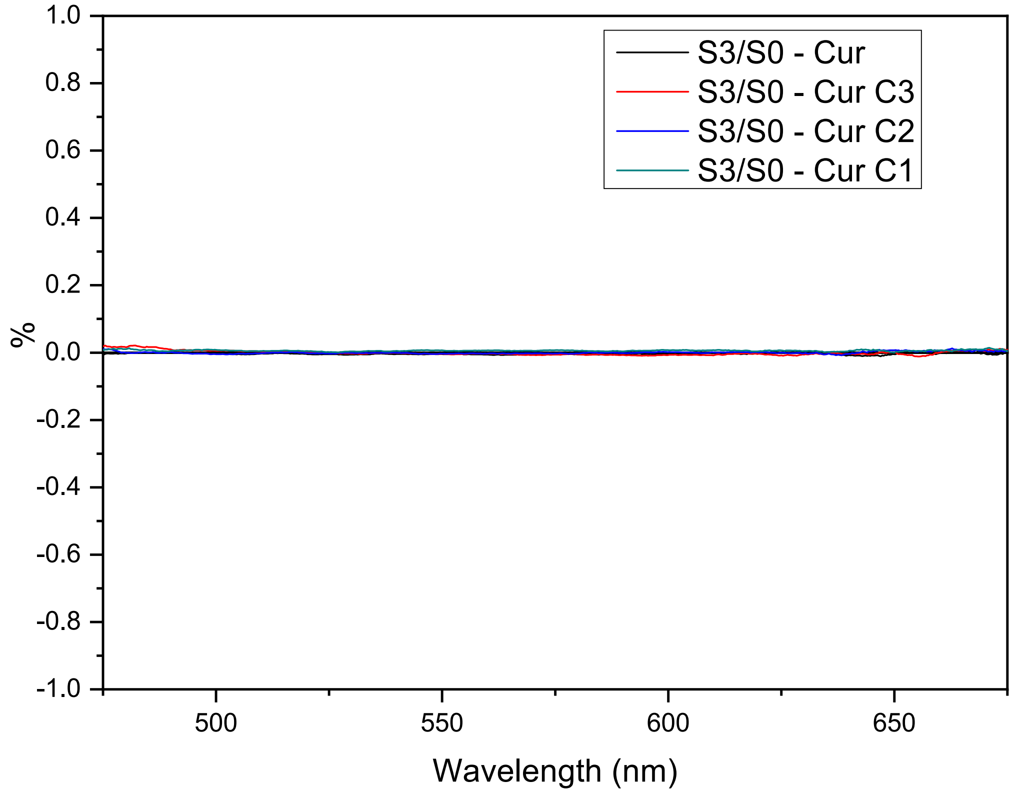

Figure SI20. S3/S0 ratio of Cur, with and without an applied magnetic field.

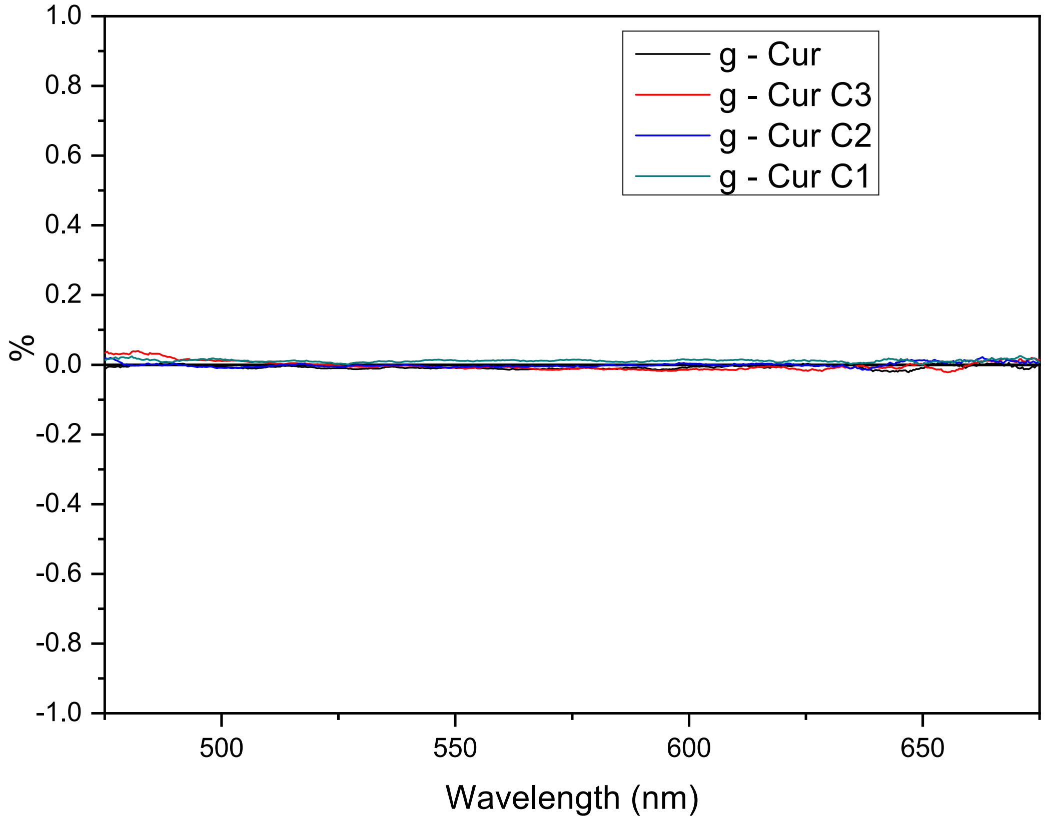

Figure SI21. Asymmetry (g) factor of Cur, with and without an applied magnetic field.

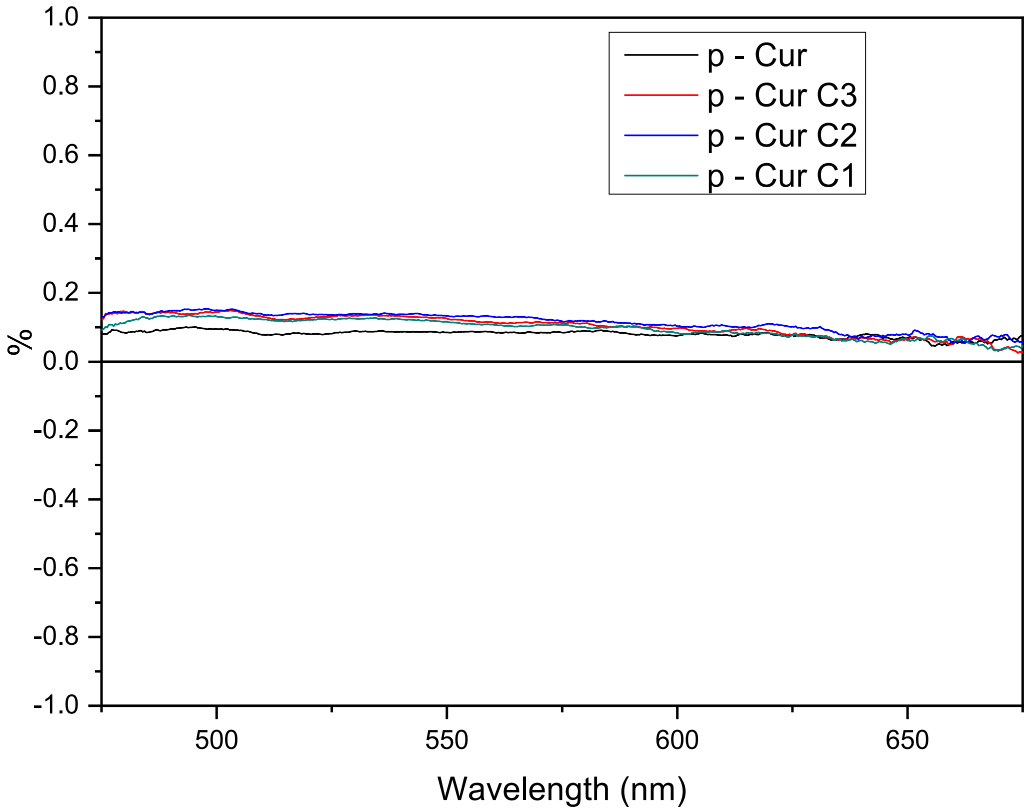

Figure SI22. Degree of polarization (p) of Cur, with and without an applied magnetic field.

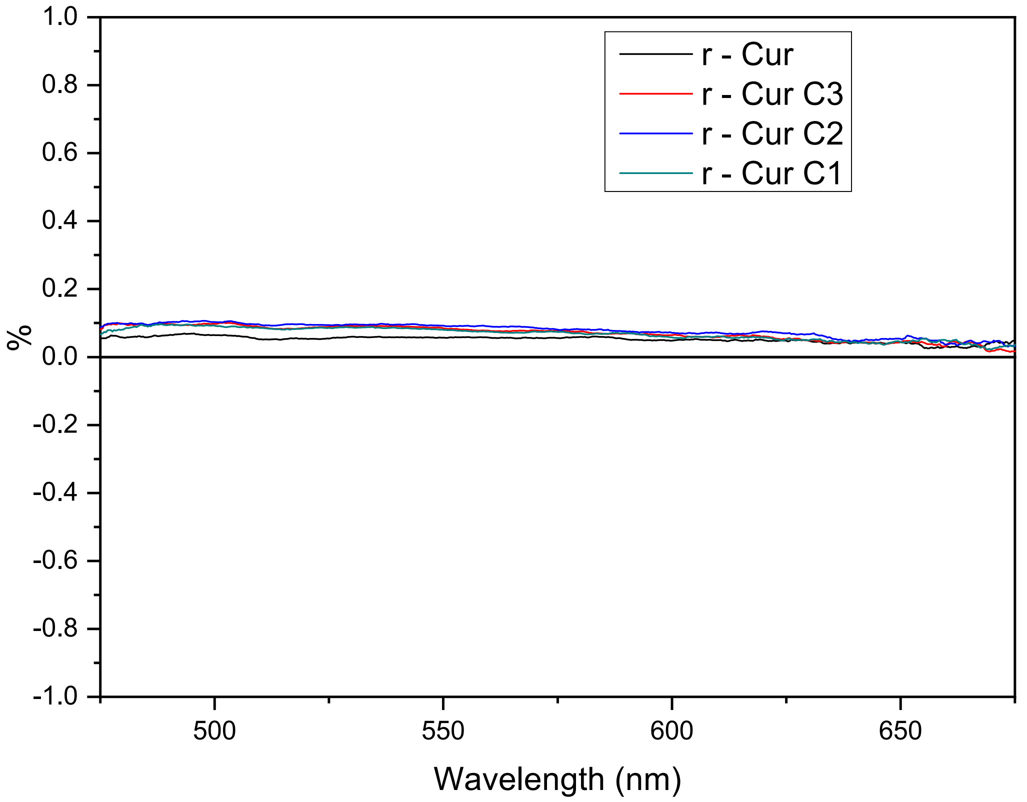

Figure SI23. Anisotropy (r) factor of Cur, with and without an applied magnetic field.

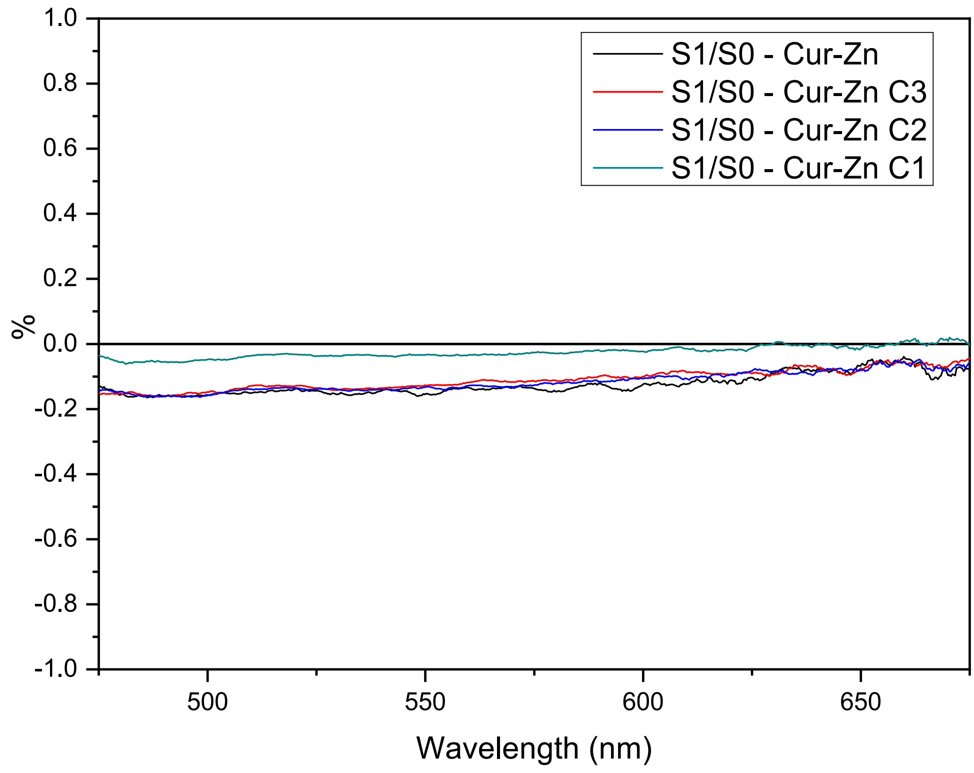

Figure SI24. S1/S0 ratio of Cur-Zn, with and without an applied magnetic field.

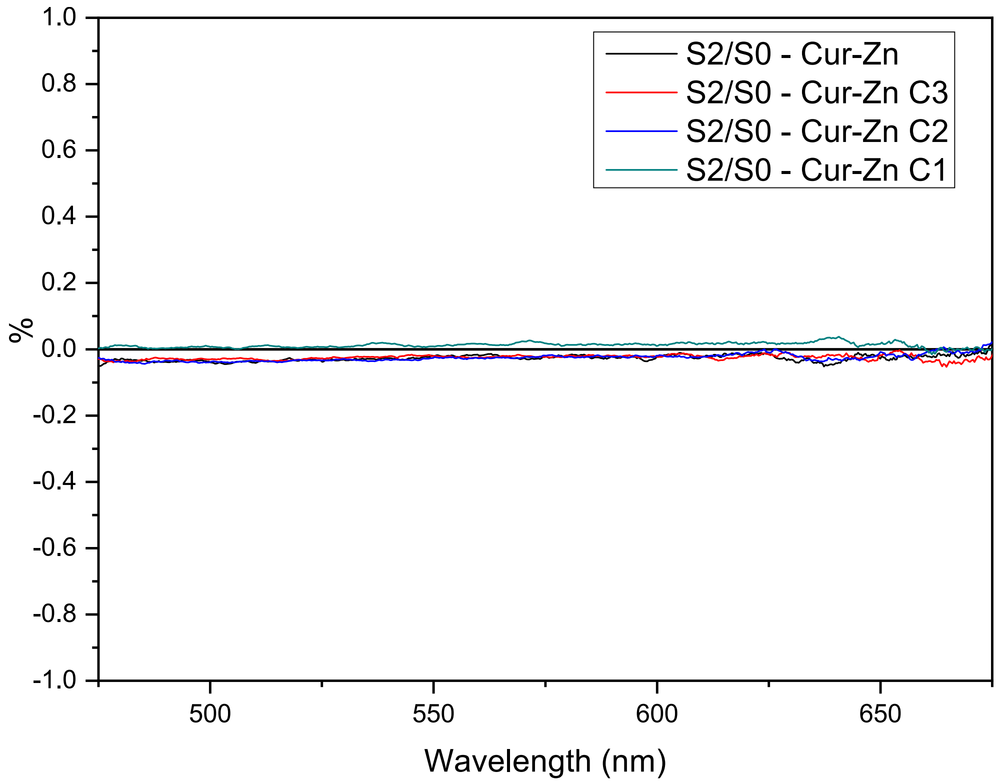

Figure SI25. S2/S0 ratio of Cur-Zn, with and without an applied magnetic field.

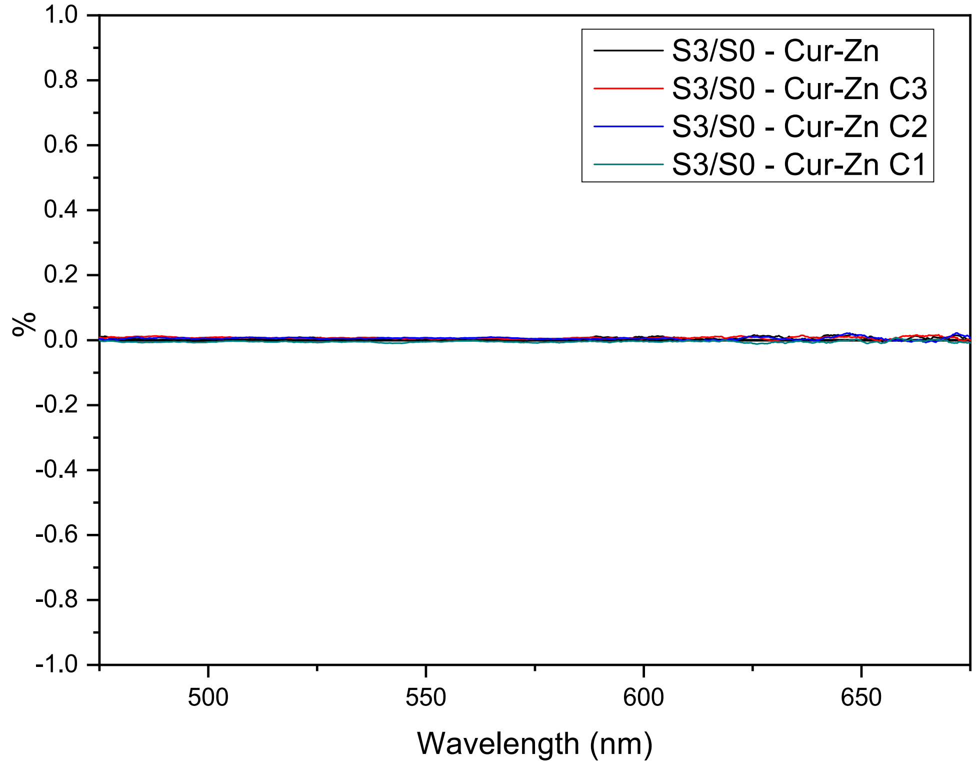

Figure SI26. S3/S0 ratio of Cur-Zn, with and without an applied magnetic field.

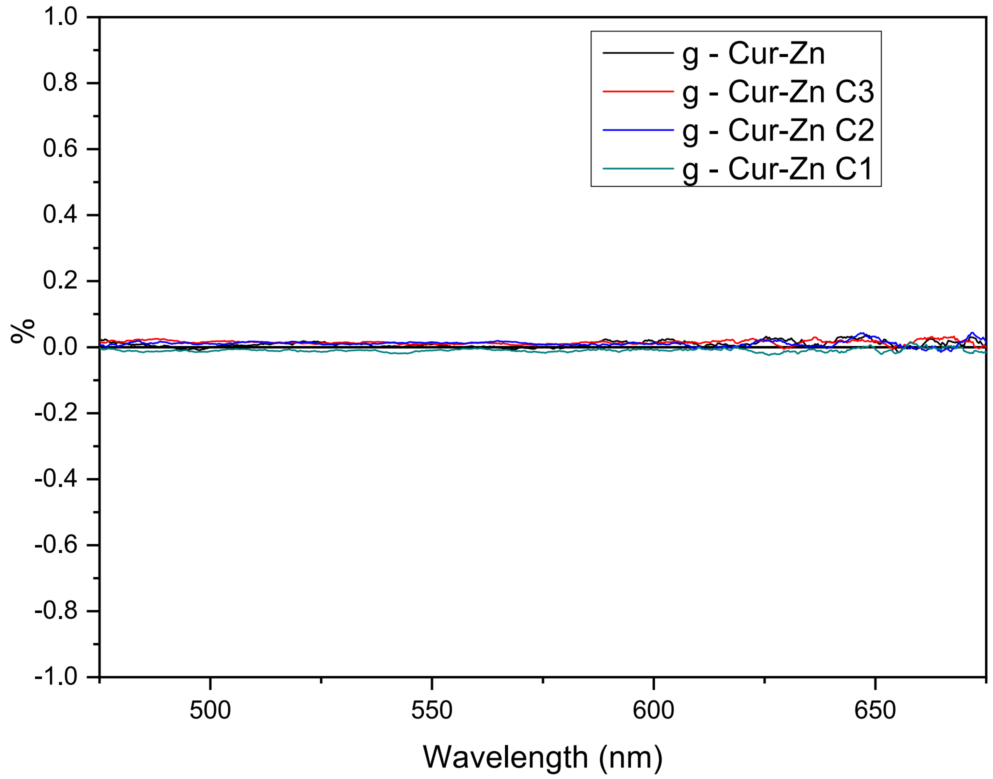

Figure SI27. Asymmetry (g) factor of Cur-Zn, with and without an applied magnetic field.

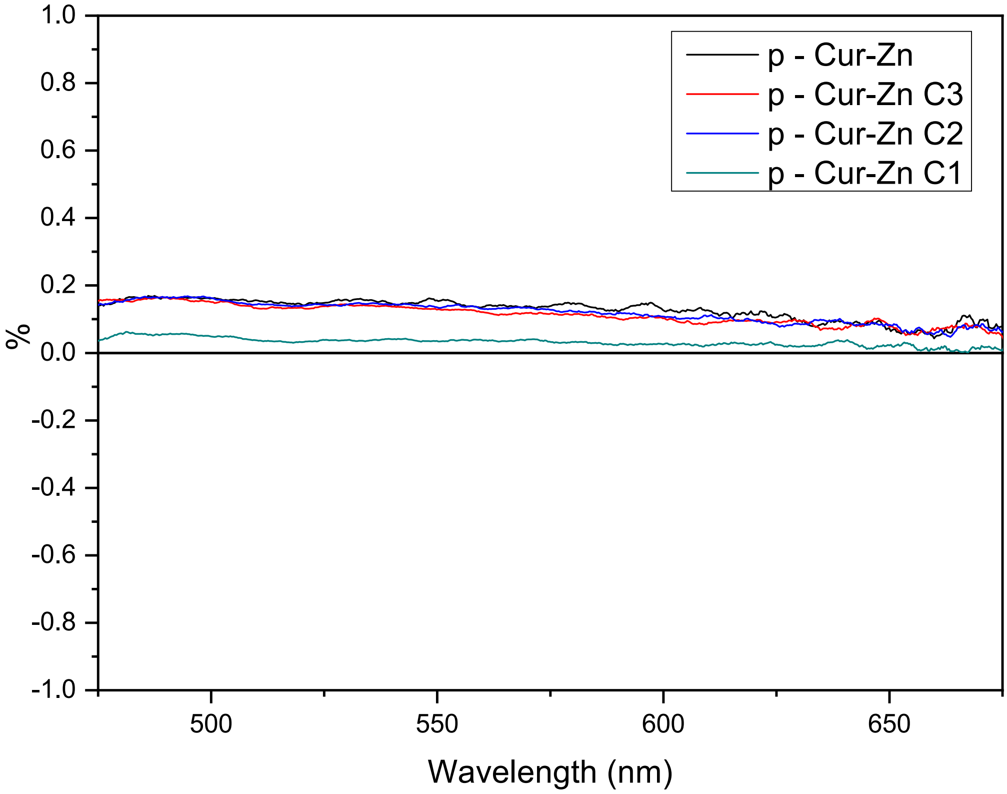

Figure SI28. Degree of polarization (p) of Cur-Zn, with and without an applied magnetic field.

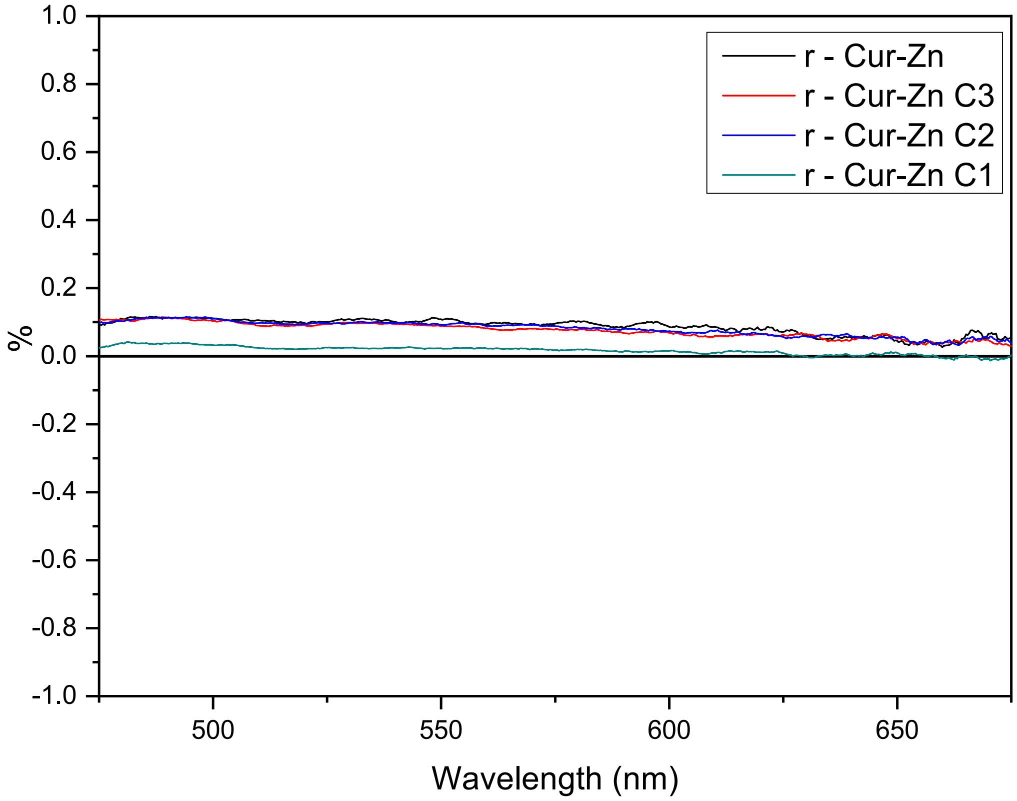

Figure SI29. Anisotropy (r) factor of Cur-Zn, with and without an applied magnetic field.

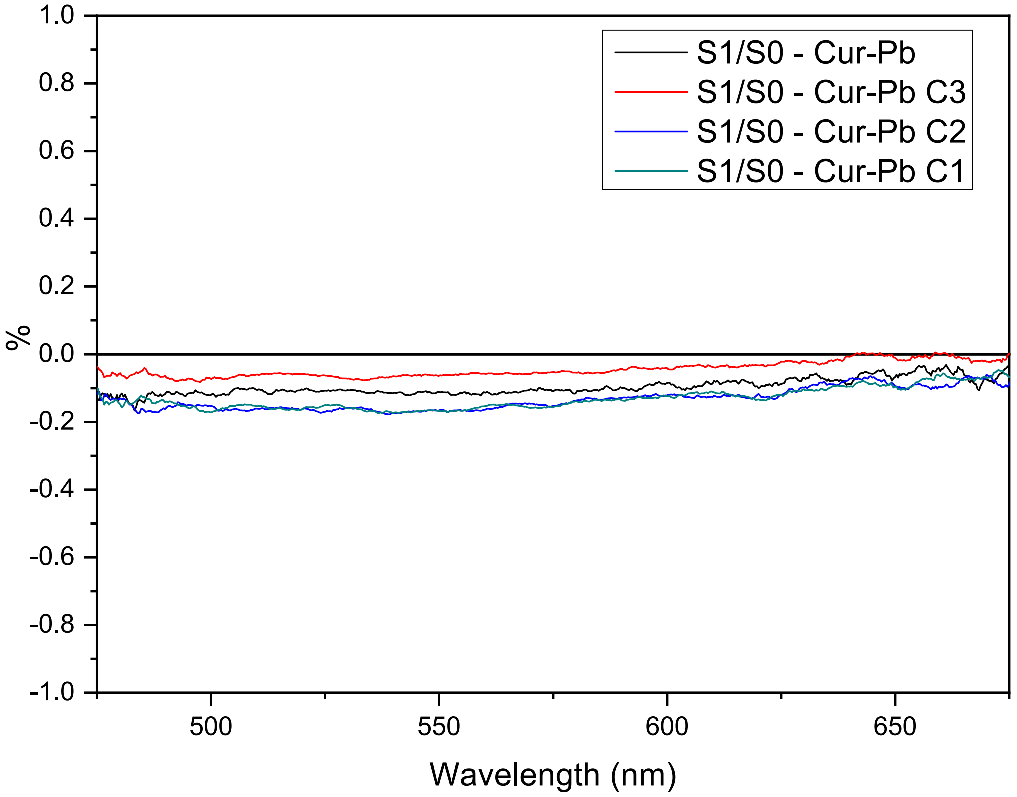

Figure SI30. S1/S0 ratio of Cur-Pb, with and without an applied magnetic field.

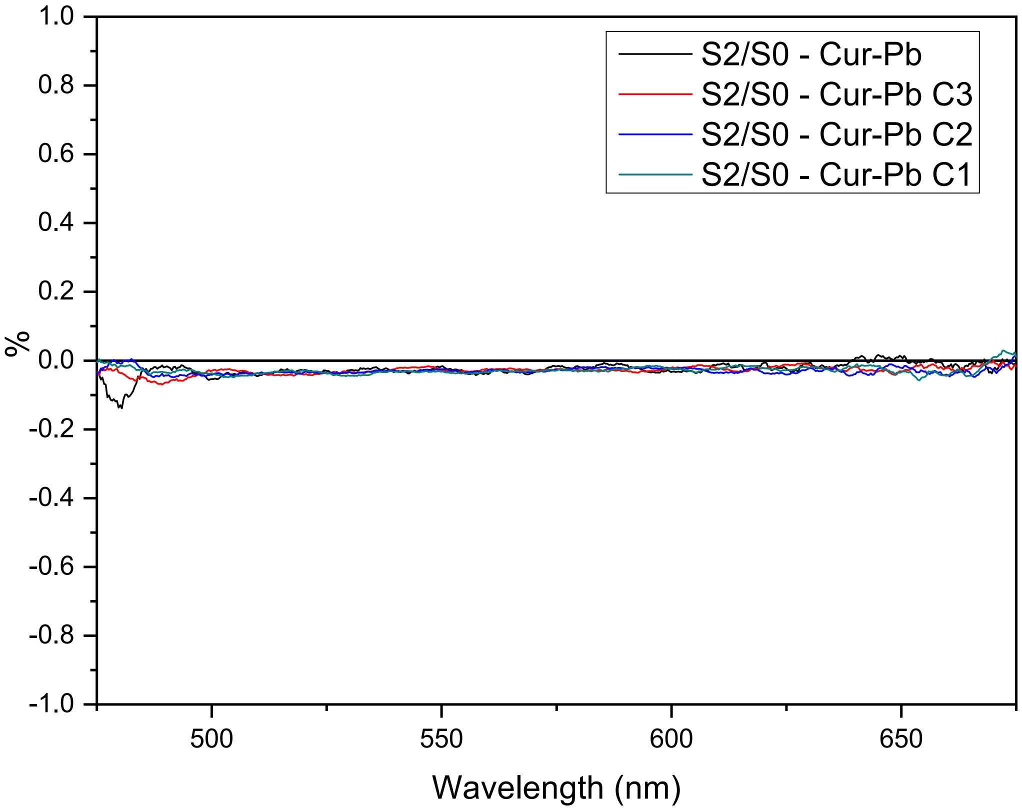

Figure SI31. S2/S0 ratio of Cur-Pb, with and without an applied magnetic field.

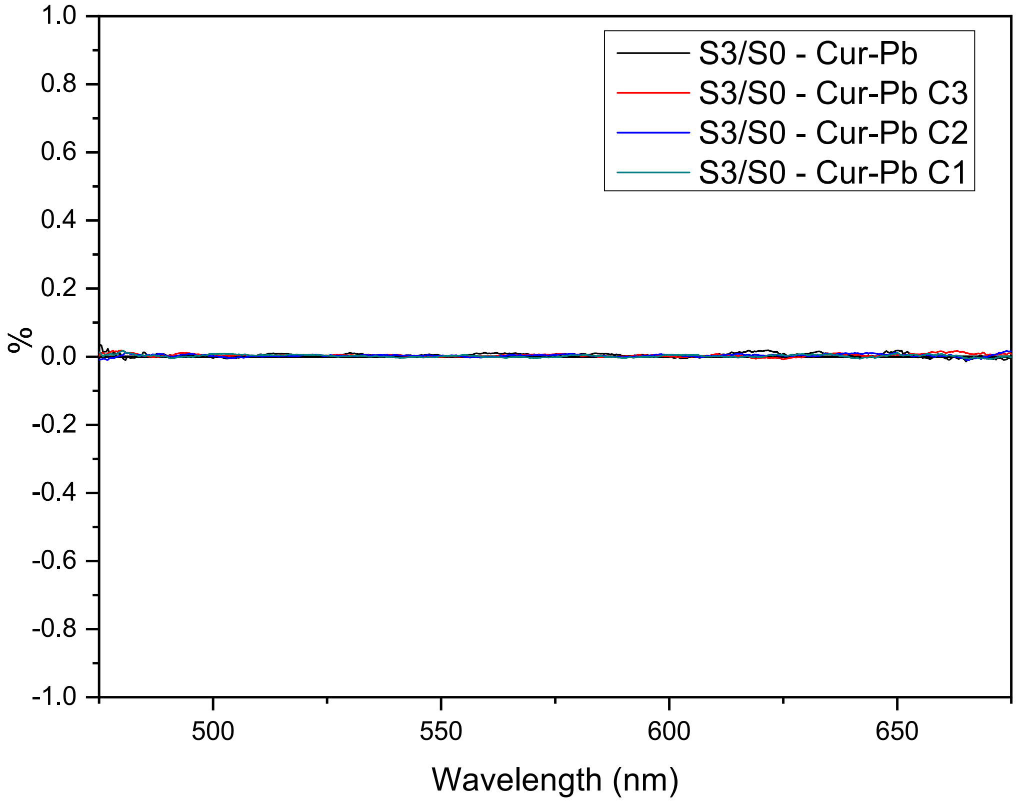

Figure SI32. S3/S0 ratio of Cur-Pb, with and without an applied magnetic field.

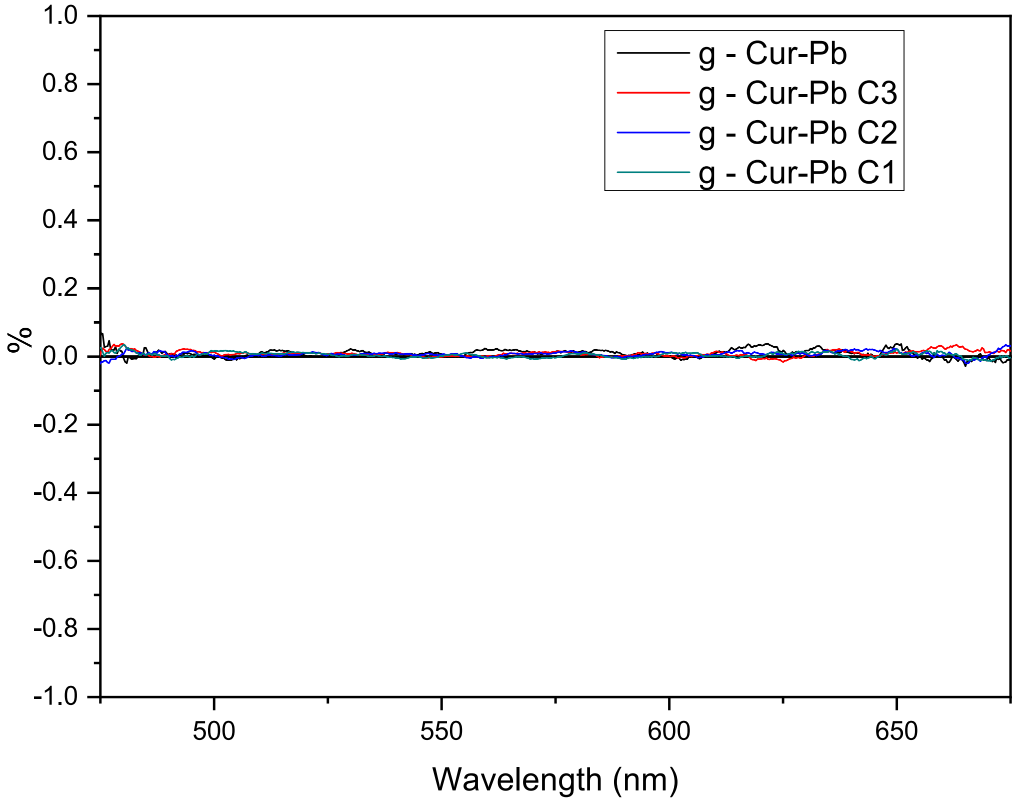

Figure SI33. Asymmetry (g) factor of Cur-Pb, with and without an applied magnetic field.

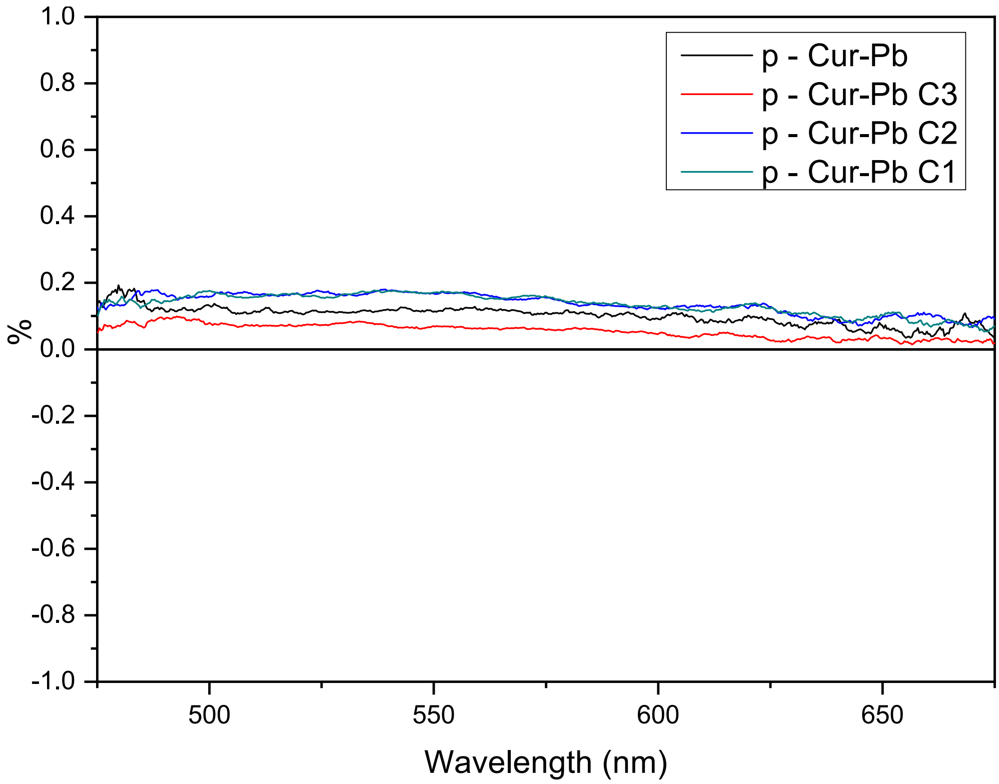

Figure SI34. Degree of polarization (p) of Cur-Pb, with and without an applied magnetic field.

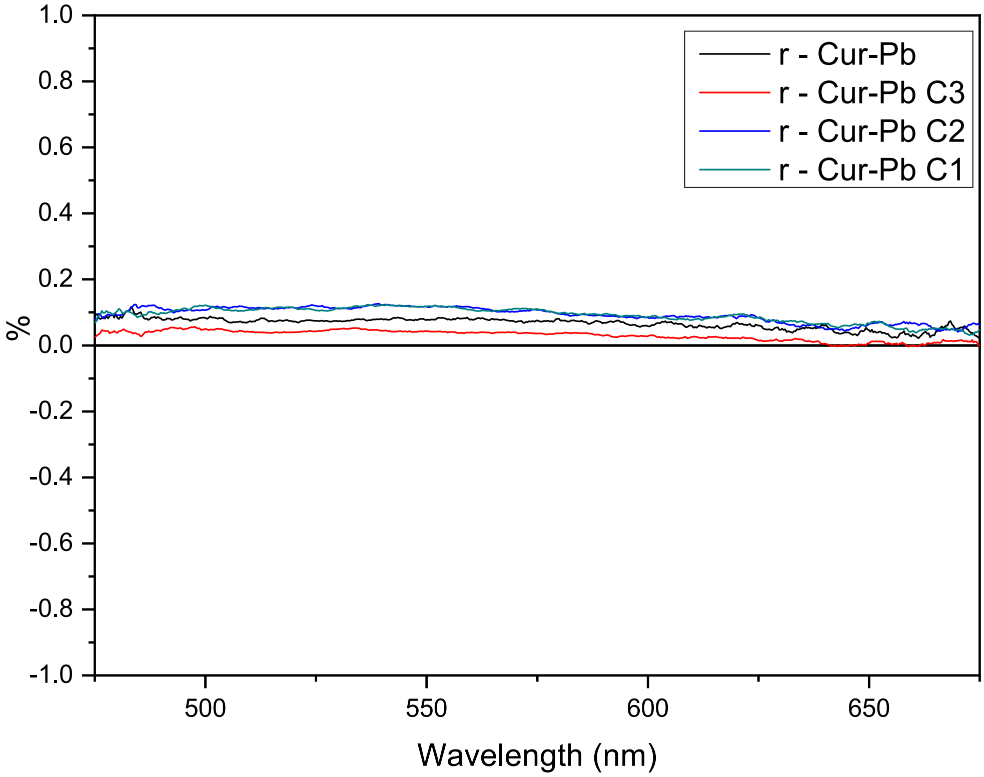

Figure SI35. Anisotropy (r) factor of Cur-Pb, with and without an applied magnetic field.

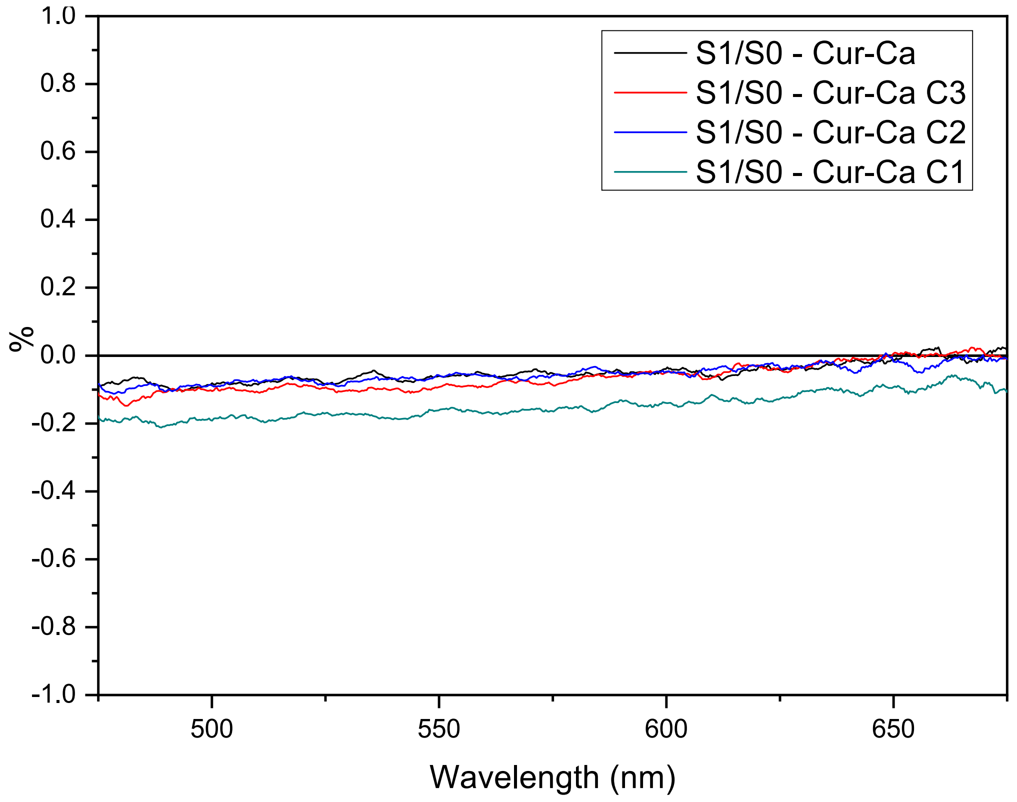

Figure SI36. S1/S0 ratio of Cur-Ca, with and without an applied magnetic field.

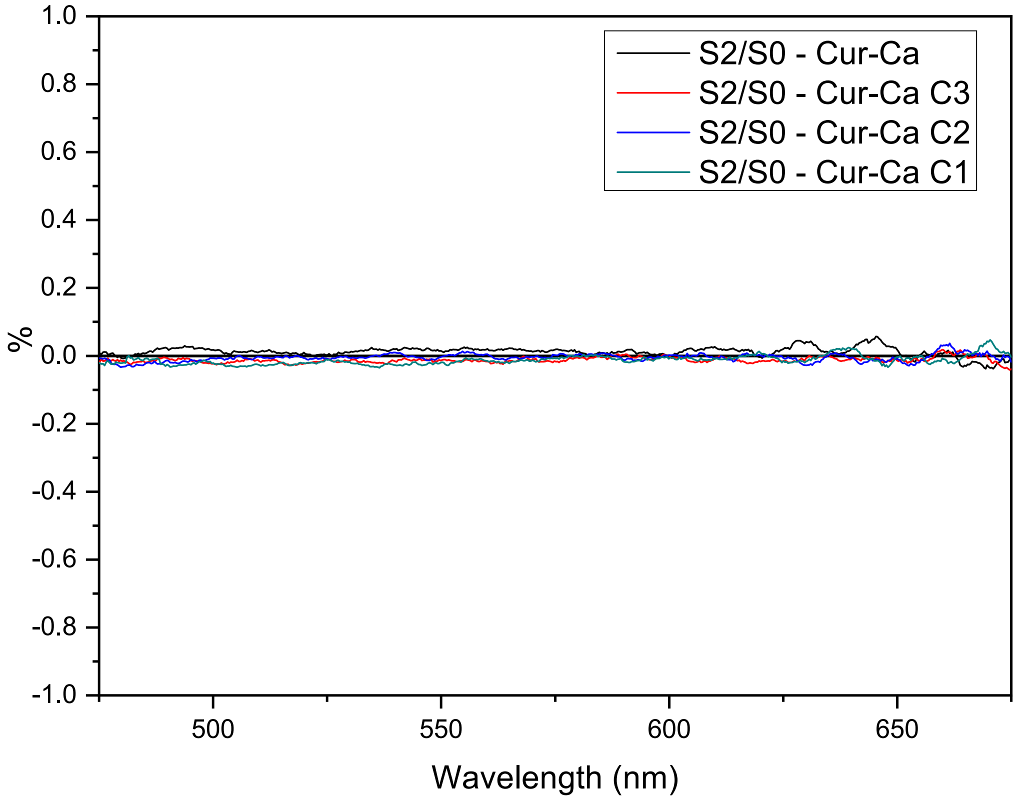

Figure SI37. S2/S0 ratio of Cur-Ca, with and without an applied magnetic field.

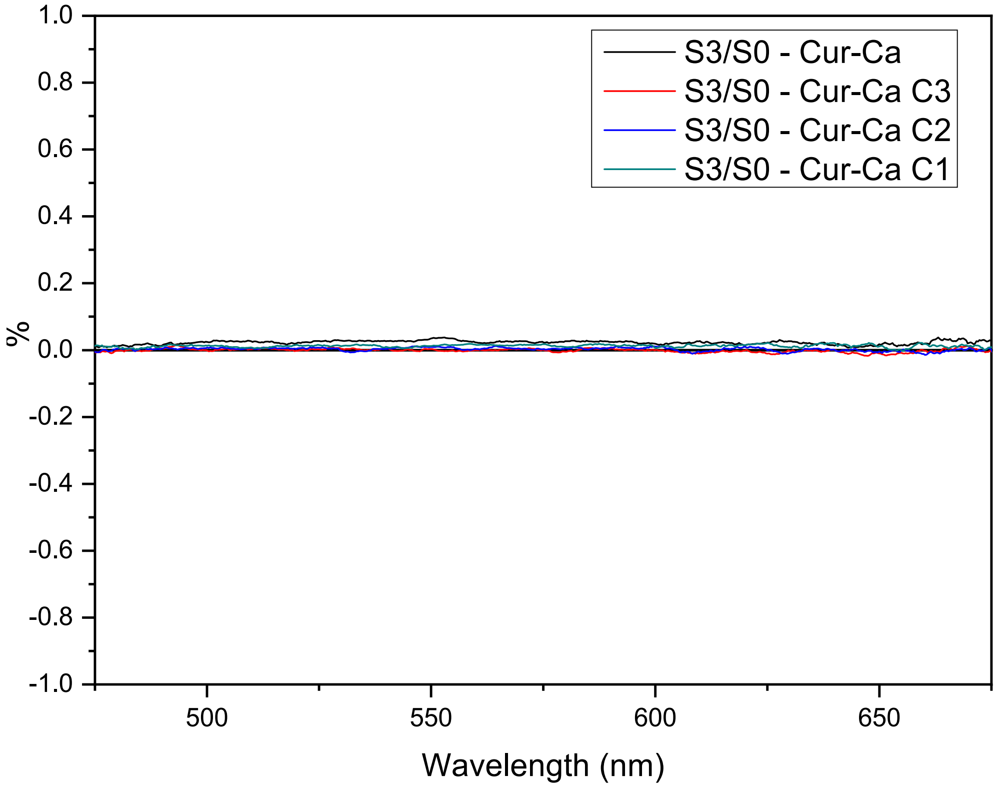

Figure SI38. S3/S0 ratio of Cur-Ca, with and without an applied magnetic field.

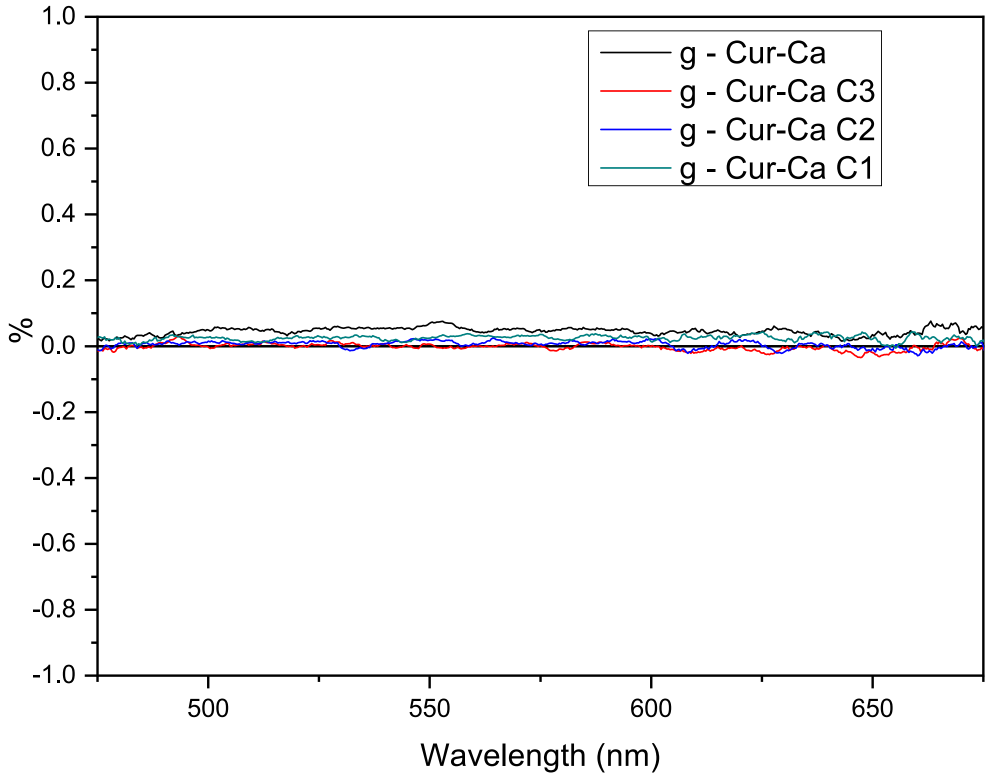

Figure SI39. Asymmetry (g) factor of Cur-Ca, with and without an applied magnetic field.

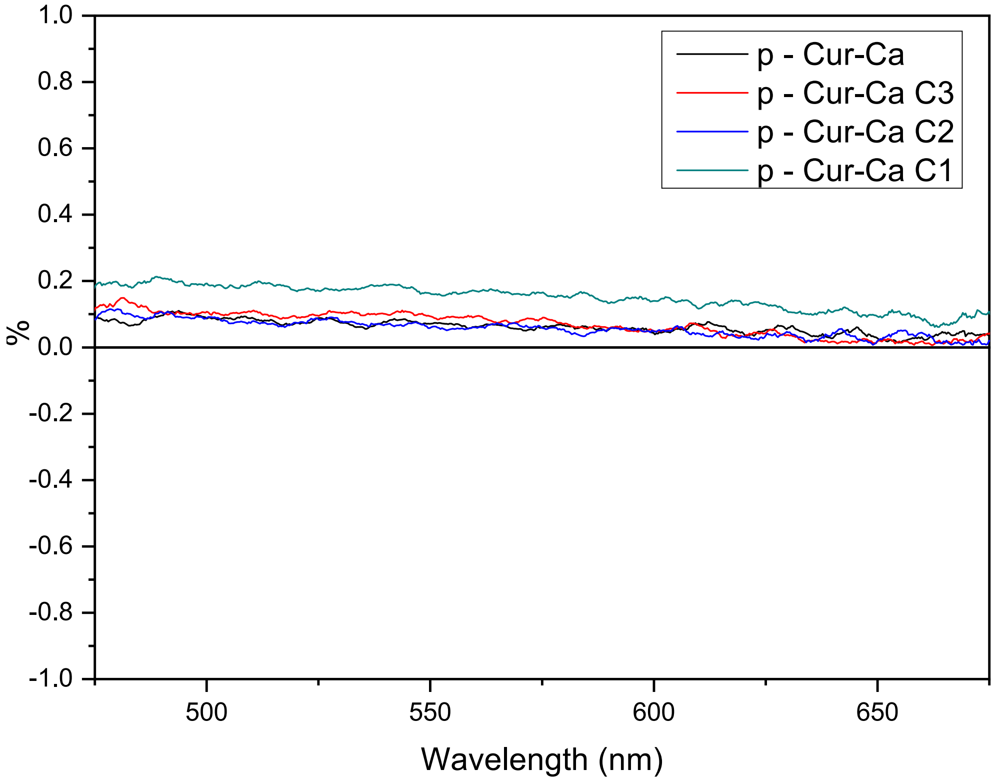

Figure SI40. Degree of polarization (p) of Cur-Ca, with and without an applied magnetic field.

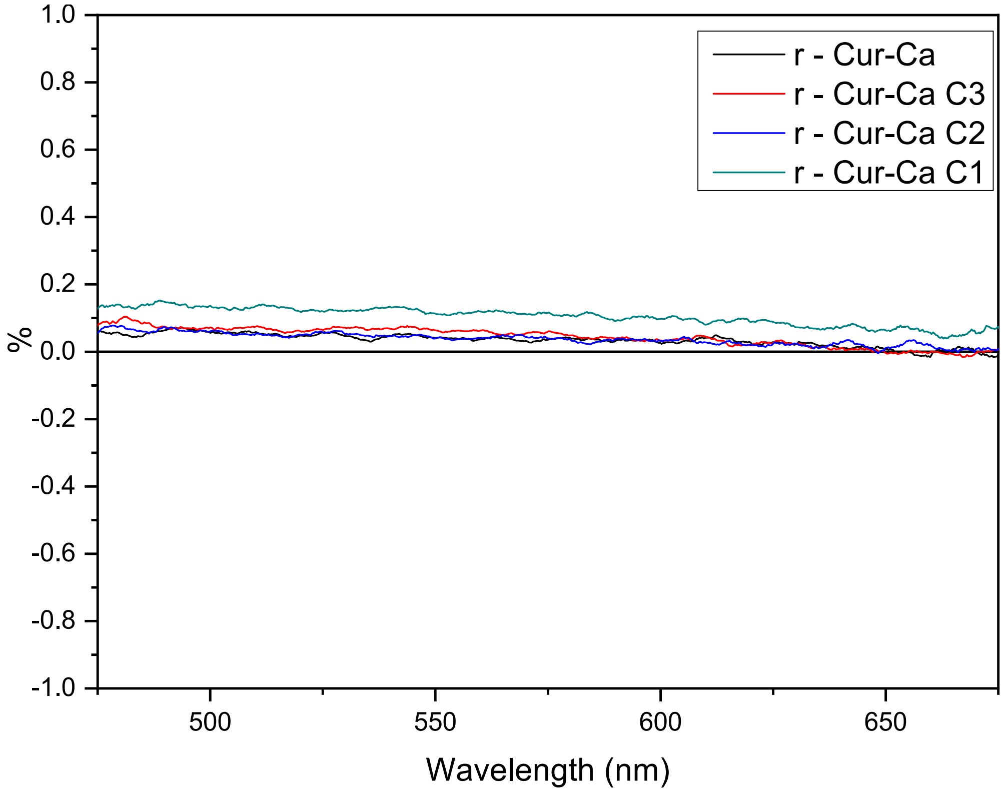

Figure SI41. Anisotropy (r) factor of Cur-Ca, with and without an applied magnetic field.

Table SI1. Emission Ellipsometry parameters of Cur, obtained with and without an applied magnetic field.

MAGNETIC FIELD (mT)	S1/S0	S2/S0	S3/S0	p	r	g
**0.00**	-0.07578	-0.01393	0.01859	0.17695	0.06910	0.03719
**0.10**	-0.10458	0.01193	0.05339	0.13731	-0.01794	0.03072
**0.20**	-0.11721	-0.00451	0.01997	0.09362	0.01732	0.03186
**0.47**	-0.09809	0.02753	0.01625	0.07516	0.00807	0.02054

Table SI2. Emission Ellipsometry parameters of Cur-Zn, obtained with and without an applied magnetic field.

MAGNETIC FIELD (mT)	S1/S0	S2/S0	S3/S0	p	r	g
**0.00**	-0.01485	0.02564	0.01549	0.19502	0.01460	0.03098
**0.10**	-0.05836	-0.00683	0.00520	0.14658	0.03860	0.01040
**0.20**	0.01210	0.01939	0.01149	0.13996	-0.00320	0.02298
**0.47**	-0.01664	0.00794	-0.02353	0.10200	0.01605	-0.04705

Table SI3. Emission Ellipsometry parameters of Cur-Pb, obtained with and without an applied magnetic field.

MAGNETIC FIELD (mT)	S1/S0	S2/S0	S3/S0	p	r	g
**0.00**	-0.06926	0.06654	0.00535	0.17869	0.00171	0.01070
**0.10**	-0.02911	0.00820	0.00901	0.10999	0.02125	0.01801
**0.20**	-0.09081	0.02691	-0.00622	0.21516	-0.04337	-0.01245
**0.47**	-0.04027	-0.01054	0.01850	0.12486	0.03472	0.03699

Table SI4. Emission Ellipsometry parameters of Cur-Ca, obtained with and without an applied magnetic field.

MAGNETIC FIELD (mT)	S1/S0	S2/S0	S3/S0	p	r	g
**0.00**	0.02131	-0.01860	-0.00690	0.22227	-0.00878	-0.01379
**0.10**	-0.01922	0.01711	0.01974	0.16597	0.02160	0.03949
**0.20**	0.30352	-0.11113	0.07342	0.14133	0.00099	0.05026
**0.47**	-0.07065	0.00825	-0.02795	0.19175	0.06135	-0.01929
